# The Effects of Online Cognitive Behavioral Therapy on Postpartum Depression: A Systematic Review and Meta-Analysis

**DOI:** 10.3390/healthcare13070696

**Published:** 2025-03-21

**Authors:** Jingyu Pan, Wenjing Luo, Haijuan Zhang, Yong Wang, Hong Lu, Chongkun Wang, Chunying Li, Li Fu, Yinchu Hu, Yuxuan Li, Meidi Shen

**Affiliations:** 1 School of Nursing, Peking University, Beijing 100191, China; pjy8116@bjmu.edu.cn (J.P.); wenjingluo@bjmu.edu.cn (W.L.); wangchongkun@stu.pku.edu.cn (C.W.); yinchuhu@bjmu.edu.cn (Y.H.); 2111110236@bjmu.edu.cn (M.S.); 2 Peking University Sixth Hospital, Beijing 100191, China; 3 Institute of Mental Health, Peking University, Beijing 100191, China; 4 NHC Key Laboratory of Mental Health, Peking University, Beijing 100191, China; 5 National Clinical Research Center for Mental Disorders, Peking University Sixth Hospital, Beijing 100191, China; 6 Health Science Library, Peking University, Beijing 100191, China; leecy@bjmu.edu.cn; 7 School of Nursing, Liaoning University of Traditional Chinese Medicine, Shenyang 110847, China; ful-hlxy@lnutcm.edu.cn; 8 School of Medicine, Tsinghua University, Beijing 100084, China; li-yx23@mails.tsinghua.edu.cn

**Keywords:** postpartum depression, cognitive behavioral therapy, online, systematic review, meta-analysis

## Abstract

Background/Objectives: Postpartum depression seriously affects the safety and health of mothers and children. Online cognitive behavioral therapy is considered to be a promising treatment; however, whether it is effective at improving postpartum depression is inconsistent and the specific intervention measures are not the same. The objectives of this study were to comprehensively review the effects of online cognitive behavioral therapy on postpartum depression and further explore the specific intervention measures. Methods: A literature search was conducted using thirteen electronic databases and two clinical trial registries from the establishment of the databases to 31 December 2023. The study selection and data extraction were independently performed by two researchers. The latest Cochrane Risk of Bias tool was selected to evaluate the quality of the included studies. Data were analyzed using Review Manager 5.4, and the certainty of the evidence was evaluated using the online GRADEpro tool. Eighteen studies involving 3689 women were included. Results: The results showed that online cognitive behavioral therapy was effective at improving postpartum depression. A subgroup analysis showed that the duration of online cognitive behavioral therapy with total intervention was 9 weeks and above, the total intervention number was 12 times or fewer, and using a website or Zoom online conference room as the intervention platform could more significantly improve postpartum depression. In particular, providing professional guidance could be more effective. Conclusions: In summary, online cognitive behavioral therapy was effective for postpartum depression. Furthermore, this study found out how specific intervention measures of online cognitive behavioral therapy could be more effective. Finally, nurses can participate in the therapy to improve access to evidence-based treatment.

## 1. Introduction

Postpartum depression (PPD) refers to depression that occurs in women within 1 year after childbirth, and is one of the most common postpartum complications [1]. The prevalence of PPD is about 17.22% worldwide, which varies from country to country [2,3,4]. PPD is very harmful and seriously affects the safety and health of women and children [1]. The mother’s work and social adaptability are weakened, the quality of life is reduced, the physical and mental health are weakened, and it can lead to chronic or chronic recurrent depression and even suicidal behavior [5]. For infants, the care behavior of mothers often significantly changes, resulting in a disconnection between mother and child, which affects the emotional, cognitive, and social development of infants [6].

At present, the treatment of postpartum depression mainly includes psychotherapy, drug therapy, physical therapy, and other treatments (such as exercise therapy and light therapy). For mild and moderate postpartum depression, structured psychotherapy (such as cognitive behavioral therapy and interpersonal psychotherapy) is recommended as the first-line treatment, and patients with severe postpartum depression are recommended to be referred to a psychiatric clinic [7].

Cognitive behavioral therapy (CBT) was first proposed by Dr Aaron Beck in 1964 [8,9]. CBT focuses on the role of cognitive activities in the occurrence or outcome of an individual’s emotional or behavioral issues, re-orienting thoughts, feelings, and behavioral responses and adjusting patterns to improve psychological outcomes and wellbeing [10]. CBT has become the most widespread and commonly used modality of psychological therapy around the world. The National Institute for Health and Care Excellence [11], the American Psychological Association [12], and the Scottish Intercollegiate Guidelines Network [13] all recommend CBT as the preferred therapy in psychotherapy. Numerous studies have established that CBT is highly effective when treating symptoms of postpartum depression, demonstrating improvement and stable outcomes over time [14,15].

There is evidence that nurses have the ability to provide CBT to women with postpartum depression in primary care and have good results, feasibility, and acceptability. Milgrom and their research team believe that nurses are the main primary health care professionals in contact with women during the postpartum period and that CBT is very suitable for nurses, which can be successfully transformed into extensive transmission by nurses [16]. A study on nurses providing CBT for women with postpartum depression found that it is feasible for nurses to provide online CBT for women with postpartum depression, showing that it could reduce the burden of depression on women and their children and improve women’s access to evidence-based postpartum depression treatment [17]. Ngai and their research team conducted a study on postpartum depression in Hong Kong, China. The results showed that the telephone-based CBT intervention program provided by professionally trained nurses had a clinically significant and statistically significant effect, and could have a significant impact on the related huge public health burden [18].

With the development of information technology and mobile health care, online cognitive behavioral therapy (OCBT) is considered to be a promising treatment for postpartum depression. OCBT uses the internet as the medium, sharing guided therapy content derived from CBT in a modular, structured way throughout a pre-defined treatment program to guide users to carry out treatment. OCBT is designed to be used with flexibility, allowing the users—especially postpartum women—to access, read, listen to, or complete certain tasks and activities at their own pace unlimited by clinic or working hours. It also incurs a lower cost. At the same time, OCBT has various forms; users can choose according to their own preferences, exerting individual autonomy. It is easier to operate and accept, and achieves the desired psychological treatment goals [19].

Although a large number of studies have recommended OCBT for the treatment of postpartum depression, whether OCBT is effective at improving postpartum depression is inconsistent. For example, the development and experimental evaluation of an internet-based cognitive behavioral intervention model for postpartum depression showed that the model had high feasibility, with low consumption, high project utilization, and high satisfaction [20]. The study by Jeannette Milgrom [21] stated that the experimental group (using a cognitive behavioral therapy website for postpartum depression) was superior to the control group at encouraging and maintaining reductions in symptom severity over a 21-week follow-up for women with symptoms of postpartum depression, and they provided direct empirical support that internet-delivered treatment for women with symptoms of postpartum depression was a viable alternative to face-to-face treatment. However, Ji-Min Seo [22] found that using a mobile application for postpartum depression self-management did not lead to any difference in postpartum depression between the experimental and control groups after an eight-week intervention.

OCBT intervention measures for postpartum depression are variable and include the intervention platform, treatment duration, treatment frequency, or whether professional support is provided. In 2020, Maria Roman published a systematic review and meta-analysis [23] that systematically searched for studies on OCBT for postpartum depression between 2000 and 2017, but did not explore the specific intervention measures in more depth. In addition, with the increasing use of the internet and teletherapy to provide various forms of treatment and the growing awareness of postpartum depression symptoms and challenges, there has been a proliferation of studies published since this systematic review was conducted to explore the impact of OCBT on postpartum depression [24,25,26,27,28,29,30,31].

In summary, the purpose of this study was to aggregate the available published evidence on online cognitive behavioral therapy for postpartum depression, assess the overall impact on the outcomes for postpartum depression, and delineate the intervention measures.

## 2. Materials and Methods

This systematic review and meta-analysis used the Cochrane Handbook for Systematic Reviews of Interventions (Version 6.4, 2023) [32] as the methodological guide and the Preferred Reporting Items for Systematic Reviews and Meta-Analyses Statement (PRISMA 2020 Checklist) [33] as the report checklist (Supplementary Table S1). The protocol was registered in advance at PROSPERO (CRD42024504488).

### 2.1. Search Strategy

The general search principle was based on the JBI three-step search strategy with the help of librarians, which included the three steps of a preliminary search, a formal search, and a supplementary search. Firstly, the researchers conducted a preliminary search using PubMed and the Chinese Biomedical Literature Service System (SinoMed) to find English and Chinese subject terms and entry terms that could identify potential target studies. Secondly, the researchers conducted a formal search using the databases, registers, and dissertation libraries and the identified English and Chinese search terms. We searched all peer-reviewed published studies in ten electronic databases (PubMed, Embase, CINAHL Plus, Web of Science, The Cochrane Library, PsycINFO, China National Knowledge Infrastructure (CNKI), WanFang Data Knowledge Service Platform (Wanfang Data), China Science and Technology Journal Database (CQVIP), and SinoMed). We then searched clinical trials from two clinical trial registration platforms (World Health Organization International Clinical Trials Registry Platform (WHOICTRP) and United States National Library of Medicine ClinicalTrials.gov (https://clinicaltrials.gov/)). We also searched unpublished grey literature in three dissertation libraries (ProQuest Dissertations & Theses Database (PQDT), Chinese Doctoral Dissertations/Master’s Theses Full-text Database (CDFD-CMFD), and China Dissertations Database (CDDB)). Thirdly, the researchers manually searched the reference lists of the included studies.

The search period extended from database inception to 31 December 2023. In order to search all relevant research evidence as comprehensively as possible, the language of publication was not restricted in the search process. The specific search strategies and results are presented in Supplementary Table S2.

### 2.2. Eligibility Criteria

#### 2.2.1. Participants

Studies involving women ≥18 years old, 0–1 year postpartum, and with postpartum depression were determined using the mental disorder diagnostic criteria or depression score scale. Women with a diagnosis of schizophrenia or bipolar disorder, severe acute mental illness (such as requiring hospitalization or involving suicidal ideation), and known substance-abuse problems were excluded.

#### 2.2.2. Intervention

Studies involving women receiving, and only receiving, a form of online cognitive behavioral therapy for postpartum depression symptoms as well as the form, platform, total duration, each duration, intervention number, module number (the number of modules set by OCBT where users could complete all modules in one intervention or one module in one intervention, which was different from the intervention number), etc., were included.

#### 2.2.3. Comparison

Conventional therapy (e.g., medication, physical therapy, or other psychotherapy) was used for a comparison.

#### 2.2.4. Outcome

The primary outcome was the change in depression score from the baseline to the endpoint, measured using a validated depression scoring system such as the Edinburgh Postnatal Depression Scale (EPDS) or Beck Depression Inventory (BDI).

#### 2.2.5. Study Design

RCTs were included. Studies that were not accessible as a full text were still included in the study proposal stage; repeated reports, those that did not describe specific interventions for online cognitive behavioral therapy, or those that did not provide measurements of depression scores before and after the intervention were excluded.

### 2.3. Study Selection

The study selection was independently conducted by two researchers. Firstly, a researcher sent all search records to Endnote 20, used the software’s duplicate search function to automatically remove duplicates, and manually removed duplicates that were not recognized by the software. Secondly, two researchers read the title and abstract of the remaining studies in turn according to the eligibility criteria. Thirdly, two researchers continued to read the full text of studies to determine if they were eventually included in this systematic review and meta-analysis. The studies that were excluded after full reading were required to have the reasons for exclusion specified. Fourthly, two researchers manually searched the references of the included studies to search for references that met the eligibility criteria. Any disagreements arising in the study selection were resolved by consensus reached through negotiation between two researchers.

### 2.4. Quality Assessment

The quality of the studies was independently assessed by two researchers using the latest version of the Cochrane Risk of Bias 2.0 tool for randomized trials with an automated Excel tool (https://www.riskofbias.info/). This tool judged the overall quality of the included studies by assessing the following five key domains of randomized controlled trial design and implementation: “bias arising from the randomization process”, “bias due to deviations from intended interventions”, “bias due to missing outcome data”, “bias in measurement of the outcome”, and “bias in the selection of the reported result”. Two researchers answered the questions in each assessment domain. According to the answers, combined with the built-in bias-risk decision-making path map, the bias risk for each assessment domain was divided into “low risk”, “some concerns”, or “high risk”. Finally, the overall risk of bias (low risk, some concerns, or high risk) for the included studies was comprehensively determined according to the evaluation results of the risk of bias for all assessment domains.

### 2.5. Data Extraction

Data extraction was also independently performed by two researchers. Two researchers pre-tested the data extraction of the first five included studies using a pre-designed initial form and finally determined a standardized form to extract the basic features and outcome data of the included studies. The standardized form included the author, country, participant details, sample size, implementation details of the intervention (e.g., time, frequency, platform, guidance, and training), the implementation details of control measures, and the evaluation details of outcomes. Two researchers cross-checked the data-extraction results and reached a consensus through discussion and negotiation. The researcher contacted the authors by email for data verification or to seek the original data for this systematic review and meta-analysis.

### 2.6. Data Analyses

Data analyses was performed using Revman 5.4 software. For dichotomous data, the risk ratios (RRs) were used to calculate the effect size and, for continuous data, the mean difference (MD) or standardized mean difference (SMD) were used. Point estimates and 95% confidence intervals (CIs) were calculated for all effect sizes, and a two-tailed *p*-value < 0.05 was considered to be statistically significant. Cochran’s Q test and Higgins’ I^2^ test were used to comprehensively evaluate the heterogeneity of the included studies. According to the results, the researcher decided whether to use the random-effect model (*p* ≤ 0.10; I^2^ > 50%) or the fixed-effect model (*p* ≥ 0.10; I^2^ < 50%). A sensitivity analysis was performed using the one-study-out method to verify the robustness and reliability of the meta-analysis aggregation results. When the number of studies included in the meta-analysis was greater than ten, a funnel plot was drawn to assess publication bias.

### 2.7. Certainty Assessment of the Body of Evidence

This study used the online GRADEpro Guideline Development tool (https://gdt.gradepro.org/ accessed on 16 December 2024) to classify the certainty of evidence. For randomized controlled trials, the initial certainty level of the evidence body is always “high”, so there are five factors that can cause the certainty level of the evidence body to be downgraded (high risk of bias, inconsistency, imprecision, indirectness, and publication bias). After importing the results of the meta-analysis into the GRADEpro tool, two researchers dynamically adjusted the certainty level of the evidence body of the outcome according to the five factors and finally made the classification (“high”, “moderate”, “low”, or “very low”).

## 3. Results

### 3.1. Search Results and Study Selection

**Figure 1:**
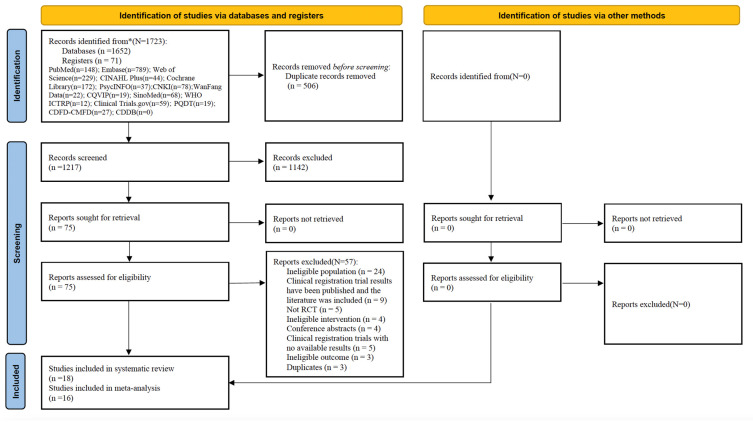
The PRISMA flow diagram. * represents the total number of records identified from databases and registers.

A total of 1723 records were searched in the databases (*n* = 1652) and registers (*n* = 71), and 506 duplicate records were removed. The remaining 1217 records were read and screened for the title and abstract, and 1142 records were excluded. The remaining 75 records were read and screened for the full text, and 57 records were excluded because they did not meet the eligibility criteria (Supplementary File S1). Finally, 18 studies [17,18,21,22,24,25,26,27,28,29,30,31,34,35,36,37,38,39] were deemed eligible and were included in the narrative synthesis; of these, 16 studies [17,21,22,24,25,26,27,28,29,30,31,34,35,36,37,38] provided enough data and were included in the meta-analysis (Supplementary File S2). The PRISMA flow diagram (Figure 1) summarizes this process.

### 3.2. Study Characteristics

Eighteen RCTs were included in this study, which were published from 2013 to 2024. The study sites included the following seven countries: Canada (*n* = 6), Australia (*n* = 3), Portugal (*n* = 3), the United Kingdom (*n* = 2), Iran (*n* = 2), China (*n* = 1), and South Korea (*n* = 1). A total of 3689 women were included in the meta-analysis, of whom 1688 received OCBT and 2001 received conventional treatment.

For the intervention measures of OCBT, seven studies had a total intervention duration of 8 weeks or fewer and eleven studies had an intervention duration of 9 weeks or longer. In terms of the total number of interventions, eleven studies had ≤8 interventions, five studies had 9–12 interventions, one study had 13–16 interventions, and one study did not mention the number of interventions. Only nine studies mentioned the duration of each intervention, which was less than 1 h (*n* = 5) and more than 1 h (*n* = 4). Of the eighteen studies, ten used a website as the delivery platform, four used application or Telegram, and four used Zoom conference rooms. The number of modules for OCBT mainly comprised ≤ 8 modules (*n* = 11), 9–12 modules (*n* = 5), or were not mentioned (*n* = 2). Of the eighteen studies, fourteen used individual interventions and four used group interventions. Professional guidance was provided in twelve studies but not in six studies. In terms of whether nurses participated in interventions, two studies mentioned nurses (midwives and public health nurses) as implementers of OCBT and sixteen studies did not. As control measures, thirteen studies implemented treatment as usual (TAU), two studies implemented a waitlist control (WLC), two studies implemented TAU and WLC, and one study implemented medication. The primary outcome measure was postpartum depression symptoms, measured using the EPDS (*n* = 15) or BDI (*n* = 3). Table 1 shows the detailed characteristics of the eighteen included studies.

### 3.3. Risk of Bias Assessment

**Figure 2:**
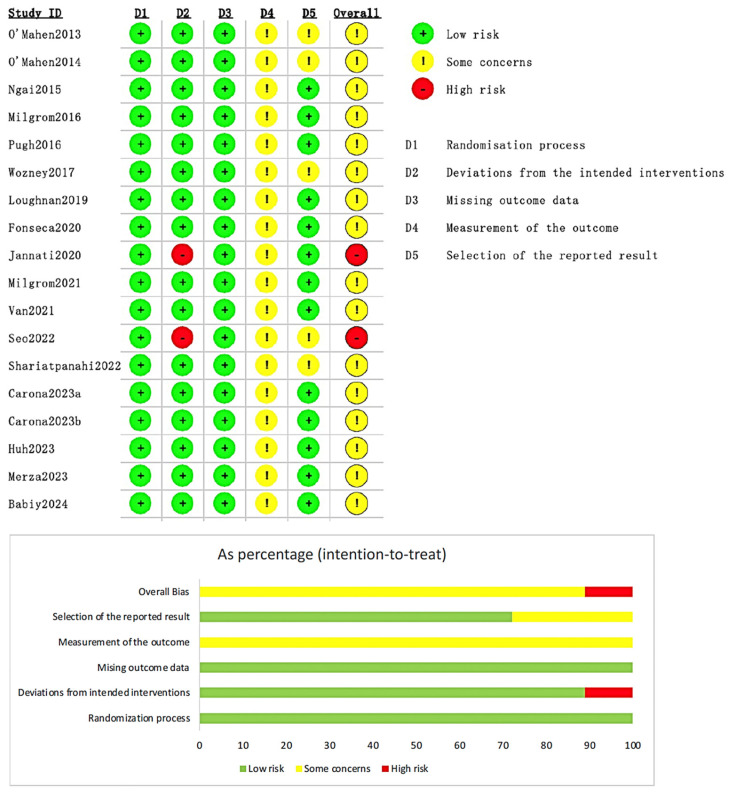
Summary of risk of bias for the eighteen studies included [17,18,21,22,24,25,26,27,28,29,30,31,34,35,36,37,38,39].

The overall results of the risk of bias assessment were within the acceptable range, and sixteen studies (*n* = 16, 88.9%) were assessed as having “some concerns” (Figure 2). Two studies (*n* = 2, 11.1%) were assessed as “high risk”, and the reason was “did not use appropriate statistical analysis methods to process the missing data”. However, the sample size of the two studies accounted for only 4.01% of the total included studies according to the relevant guideline of the Cochrane Handbook for Systematic Reviews of Interventions [32], so the two studies were eventually included in the systematic review and meta-analysis. The detailed assessment is listed in Supplementary Table S3.

### 3.4. Effects of Online Cognitive Behavioral Therapy on Postpartum Depression Outcomes

In this study, the measurement scale used was selected as the outcome indicator, and a subgroup analysis was conducted according to cultural (country) differences, intervention measures, and whether there was professional guidance. The results are as follows.

#### 3.4.1. Overall Postpartum Depressive Symptoms

**Figure 3:**
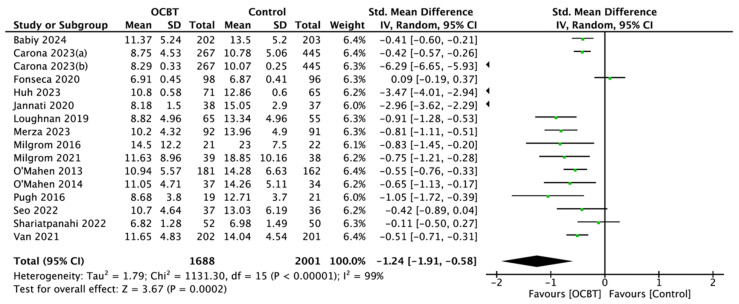
Forest plot of the effect of online cognitive behavioral therapy (OCBT) on postpartum depression symptoms [17,21,22,24,25,26,27,28,29,30,31,34,35,36,37,38].

Sixteen studies involving 3689 women evaluated the effect of OCBT on postpartum depression symptoms. The mean standard deviation (SMD) was selected as the effect indicator, and the random-effect model was selected for the meta-analysis (SMD = −1.24, 95%CI: [−1.91, −0.58], and *p* < 0.001; Figure 3).

#### 3.4.2. Subgroup Analysis of Postpartum Depressive Symptoms

##### Culture

**Figure 4:**
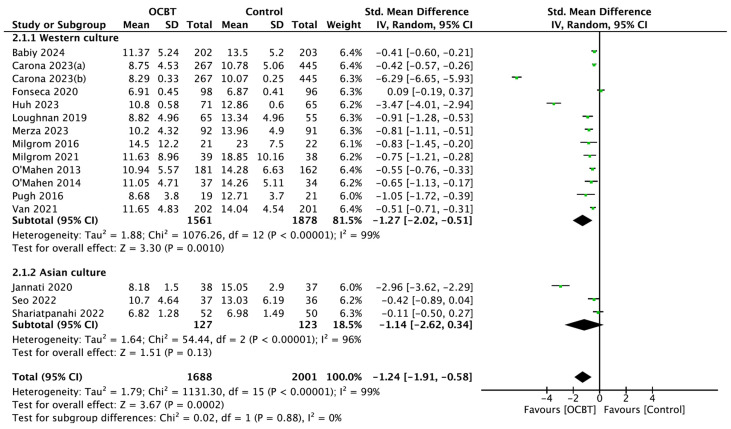
Forest plot of the effect of OCBT on postpartum depression symptoms for different cultures [17,21,22,24,25,26,27,28,29,30,31,34,35,36,37,38].

Sixteen studies assessed the effect of OCBT on postpartum depression symptoms for different cultures, including thirteen Western cultures and three Asian cultures. The meta-analysis using the random-effect model showed that OCBT treatment in Western cultures (SMD = −1.27, 95%CI: [−2.02, −0.51], and *p* = 0.001) was more effective at improving postpartum depression symptoms than in Asian cultures (SMD = −1.14, 95%CI: [−2.62,0.34], and *p* = 0.13; Figure 4).

##### Intervention Duration

**Figure 5:**
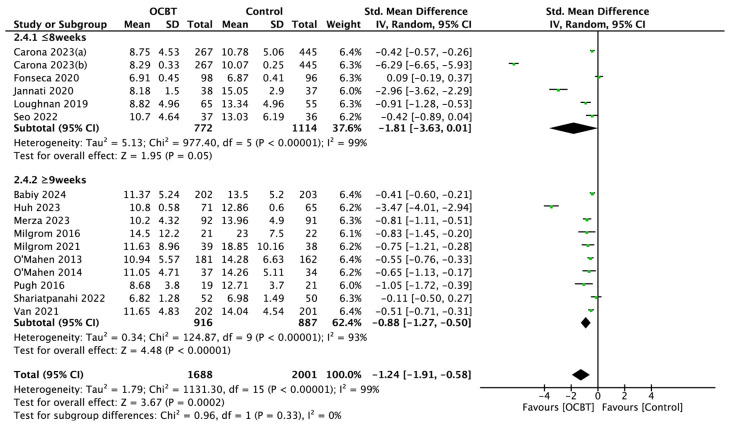
Forest plot of the effect of OCBT on postpartum depression symptoms for different intervention durations [17,21,22,24,25,26,27,28,29,30,31,34,35,36,37,38].

A total of sixteen studies evaluated the effect of different intervention durations of OCBT on postpartum depressive symptoms. The random-effect model was used for the subgroup analysis, and the pooled results showed that OCBT with intervention durations longer than 9 weeks could effectively improve postpartum depression symptoms (SMD = −0.88, 95%CI: [−1.27, −0.50], and *p* < 0.001), while OCBT with intervention durations of 8 weeks or fewer had no significant effect on postpartum depression symptoms (SMD = −1.81, 95%CI: [−3.63,0.01], and *p* = 0.05; Figure 5).

##### Intervention Number

**Figure 6:**
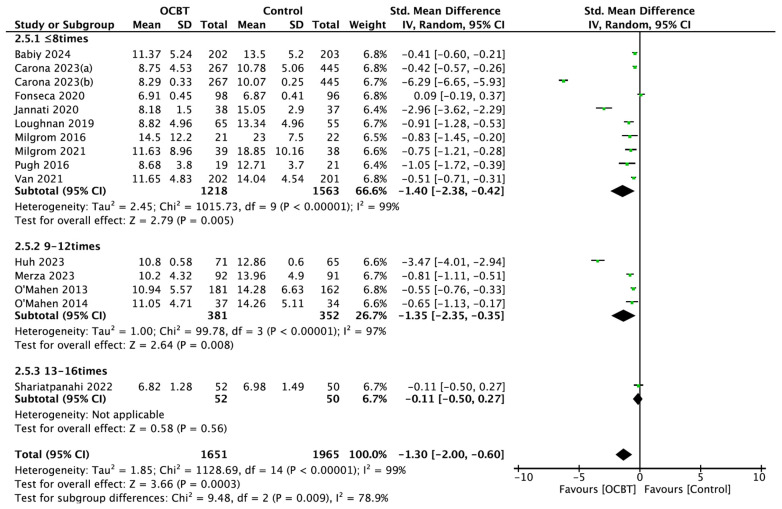
Forest plot of the effect of OCBT on postpartum depression symptoms for different intervention numbers [17,21,24,25,26,27,28,29,30,31,34,35,36,37,38].

Fifteen studies assessed the effects of OCBT with different intervention numbers on postpartum depression symptoms. The subgroup analysis showed that OCBT with fewer than 8 interventions (SMD = −1.40, 95%CI: [−2.38, −0.42], and *p* = 0.005) or between 9 and 12 interventions (SMD = −1.35, 95%CI: [−2.35, −0.35], and *p* = 0.008) could significantly improve postpartum depression symptoms, while OCBT with a total of 13–16 interventions had no significant effect on the symptoms of postpartum depression (SMD = −0.11, 95%CI: [−0.50,0.27], and *p* = 0.56; Figure 6).

##### Each Intervention Duration

Eight studies explored the effects of OCBT with different intervention durations on postpartum depression symptoms. The results of the subgroup analysis further demonstrated that an intervention duration of less than one hour or more significantly improved postpartum depression symptoms (*p* < 0.05; Supplementary Figure S1).

##### Intervention Module Number

Fourteen studies explored the effects of OCBT with different intervention module numbers on postpartum depression symptoms. The results of the subgroup analysis showed that fewer than 8 intervention module numbers or between 9 and 12 intervention module numbers could improve postpartum depression symptoms (*p* < 0.05; Supplementary Figure S2).

##### Intervention Platform

**Figure 7:**
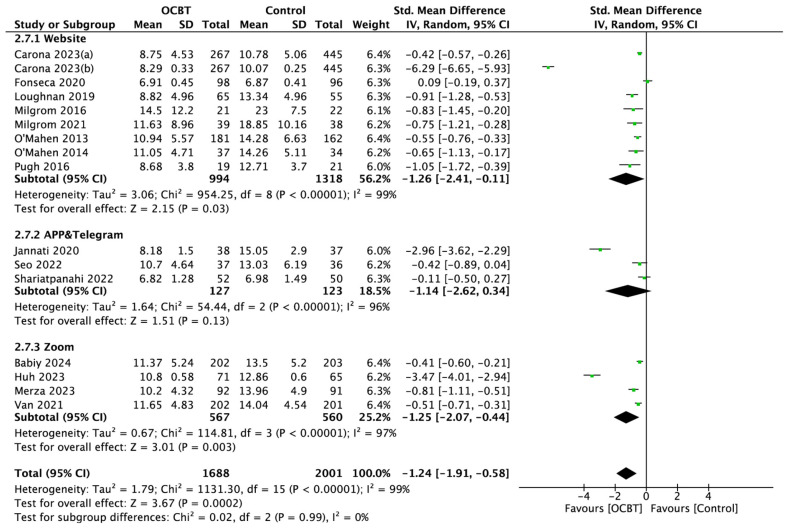
Forest plot of the effect of OCBT on postpartum depression symptoms for different intervention platforms [17,21,22,24,25,26,27,28,29,30,31,34,35,36,37,38].

According to the subgroup analysis, the pooled results showed that OCBT using a website or Zoom as the intervention platform could significantly improve postpartum depression symptoms (*p* < 0.05), while the meta-analysis of the results of three studies using an application (APP) as an intervention platform showed that OCBT using an application (APP) as an intervention platform had no significant effect on postpartum depression symptoms (SMD = −1.14, 95%CI: [−2.62,0.34], and *p* = 0.13; Figure 7).

##### Intervention Form

Sixteen studies evaluated the effects of different intervention forms of OCBT on postpartum depression symptoms. Th subgroup analysis showed that both personal and team OCBT improved postpartum depression symptoms (*p* < 0.05; Supplementary Figure S3).

##### Professional Guidance

**Figure 8:**
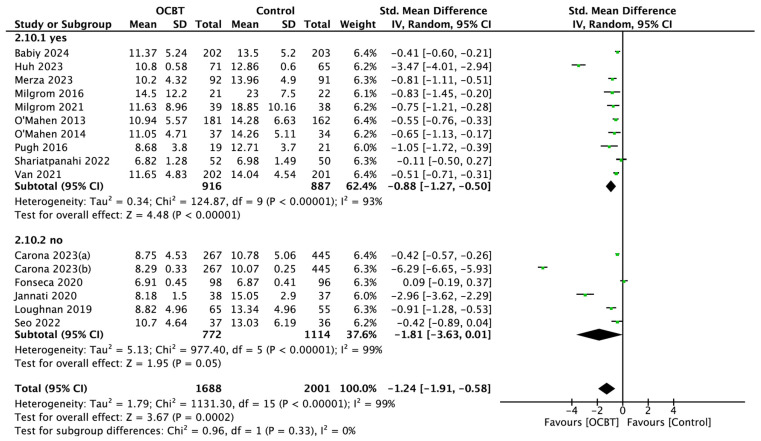
Forest plot of the effect of OCBT on postpartum depression symptoms for different intervention guidance methods [17,21,22,24,25,26,27,28,29,30,31,34,35,36,37,38].

Sixteen studies explored the effect of OCBT on postpartum depression symptoms with or without professional guidance. The pooled results showed that OCBT with professional guidance could effectively improve postpartum depression symptoms (SMD = −0.88, 95%CI: [−1.27, −0.50], and *p* < 0.001), while OCBT without professional guidance had no significant effect on postpartum depression symptoms (SMD = −1.81, 95%CI: [−3.63,0.01], and *p* = 0.05; Figure 8).

### 3.5. Sensitivity Analysis and Assessment of Publication Bias

The sensitivity analysis results showed that, after excluding a single study, there was no significant change in the pooled results, suggesting that the results of the meta-analysis were robust and reliable (Supplementary Table S4). Supplementary Figure S4 shows the funnel plots. A general observation revealed that most of the funnel plots were not completely symmetrical, suggesting the possibility of publication bias. Therefore, the researchers manually tracked the funding sources and found that, of the sixteen studies, two studies mentioned “no funding source” and the remaining fourteen studies were funded by regional development funds, national research projects, university research projects, or clearly mentioned “The funding bodies had no role in data analysis or in the decision to publish the results of the trial”.

### 3.6. Certainty of the Body of Evidence

For the certainty of the body of evidence, the overall outcome (postpartum depression symptom) was assessed as “low”. The subgroup analysis of the outcome showed that, out of eight subgroups, the body of evidence for four subgroups (intervention number, each intervention duration, intervention module number, and intervention form) was “low”. Each of the remaining four subgroups (culture, intervention duration, intervention platform, and professional guidance) was assessed as “very low”. The main reasons were inconsistency, publication bias, and imprecision. Supplementary Figure S5 shows the details of the entire evaluation process for the certainty of the body of evidence.

## 4. Discussion

This systematic review found the benefits of online cognitive behavioral therapy for women with postpartum depression, which could significantly alleviate their depressive symptoms. At the same time, a subgroup analysis was carried out according to the intervention measures. Specifically, an intervention duration of 9 weeks and above, an intervention number of 12 times or fewer, and using a website or Zoom online conference room as the intervention platform could significantly improve postpartum depression symptoms. In particular, in the implementation of OCBT, providing professional guidance could more effectively improve postpartum depressive symptoms. However, the current evidence could not prove the effect of each intervention duration on postpartum depression symptoms.

### 4.1. Interpretation of Findings

Compared with its offline face-to-face form, the core of OCBT does not change and, because of its flexibility, privacy, accessibility, and economy, it has become a major possibility for women with postpartum depression to obtain mental health support and increase their access to opportunities for treatment [40,41]. This study found that OCBT was effective at improving postpartum depression symptoms compared with conventional treatment. This was in line with previous systematic review studies of the area, which mentioned that OCBT had a good effect on improving postpartum depression symptoms, perinatal depression symptoms, common mental disorders, and subsequent sickness absence [23,42,43]. At the same time, our findings also answered questions from existing studies, including “the overall effect of OCBT on postpartum depression outcomes was not the same”. With postpartum depression becoming one of the major maternal and child health challenges in the world, the results of this study suggest promoting the implementation of OCBT by health care providers to supplement the current relative mental health support and resources available to women with postpartum depression.

Our study showed that OCBT treatment in Western cultures was more effective at improving postpartum depression symptoms than in Asian cultures. Initially, there was a greater focus on physical health, resulting in perinatal mental health being a significantly underestimated determinant of maternal health [44]. Countries in Western cultures such as the United Kingdom and Canada realized the importance of perinatal mental health earlier, leading to investment in integrated specialist services [45]. A bibliometric analysis showed that from 2000 to 2020, the United States had the largest number of published papers, which indicated its dominant position in the field of postpartum depression [46]. However, in some countries with Asian cultures, despite making significant progress in mental health services, there are still gaps in the provision of perinatal mental health [47,48].

In 2022, the NICE guideline on “Depression in adults: treatment and management” indicated that, for less severe depression, CBT should usually consist of 8 regular sessions, although additional sessions may be needed for people with comorbid mental or physical health problems or complex social needs, or to address residual symptoms [49]. As this study found, OCBT with an intervention duration of nine weeks or longer and an intervention number of twelve or fewer significantly improved postpartum depression symptoms. Firstly, the course of postpartum depression can last up to two years, so psychological treatment cannot be like medicine, which can quickly take effect within two weeks. So, as the first-line treatment of postpartum depression, psychological intervention is effective and long-term [50]. Secondly, all OCBTs are developed according to the basic steps of conventional CBT, involving mood examinations, feedback results, finding problems, setting goals, carrying out education, agreeing tasks, summarizing feedback, consolidating treatment, and other links [40]; therefore, a short-term intervention cannot fully cover all the treatment cores and the effect may be discounted. At the same time, this study also found that OCBT with a total of 13–16 intervention numbers had no significant effect on postpartum depression symptoms. Postpartum women have to face a series of problems such as childcare, so too many interventions may lead to a decline in their compliance, thus weakening the intervention effects of OCBT. As observed by Dear [51] and O’Mahen [36], in order to increase the user-friendliness of courses and reduce the onerous burden on new mothers, the research teams shortened the intervention courses to 5 or 11 sessions, respectively, which was also consistent with our subgroup analysis.

This study concluded that OCBT with professional guidance could be more effective, which was consistent with the results of Ying Lau [52]. OCBT with professional guidance provides a higher level of human support in improving depressive symptoms; it not only improves compliance, but also promotes the positive effects. Although studies have shown that unsupervised OCBT is comparable with supervised OCBT in improving anxiety and depression, the adherence is lower [53,54]. In this systematic review, using a qualitative synthesis, we summarized that the professionals included clinical psychologists, qualified psychological wellbeing practitioners, experienced midwifes who had undergone training, public health nurses, and trained peer facilitators, who provided professional guidance on OCBT intervention implementation, safety supervision, and reminder recording, and who interacted with women with postpartum depression to answer their questions during the intervention process.

CBT is usually offered by psychologists or psychiatrists; however, in this systematic review, two studies showed that it was feasible and efficient for nurses to provide OCBT to women with postpartum depression. Trained midwives and public health nurses as implementers of interventions could participate in OCBT for postpartum depression to reduce the burden of depression on women and their children while improving the symptoms of postpartum depression [17,18]. Trained nurses probably provide equal or possibly even better quality of care compared with primary care doctors, and probably achieve equal or better health outcomes for patients [55]. A nurse-led mHealth and depressive symptoms study showed that most subjects successfully participated in nurse-led mhealth interventions and that mental health nurses should play a key role in personalized mhealth activities [56]. The study by Jeannette Milgrom argued that nurses are the primary care professionals who come into contact with mothers during the postpartum period, and that effective and structured psychological interventions can be successfully translated into broad delivery by nurses. CBT is well-suited to being delivered by nurses, which could become a valuable resource for health systems around the world [16]. We believe that if nurses could be trained in OCBT, shifting the task from specialists to specialists with less specialized training, it would provide a time-friendly and unrestricted intervention for the mental care and primary care of postpartum women, improve access to evidence-based treatment, and effectively address the shortage of and imbalance in mental health resources.

### 4.2. Limitations

There are some limitations to this study. Firstly, although the researchers developed the search strategy with the help of librarians to explore relevant studies as comprehensively and accurately as possible, there may have been newer studies published within the space of time between the actual conducting of this review and the peer publication date. Secondly, upon the discussion of our research team and reference to the Cochrane Handbook, two studies that were assessed as “high risk” in the quality assessment were included in this systematic review. Thirdly, some of the certainty of evidence was rated as “low” or “very low”, and in psychological treatment or teletherapy there are some issues that require insight or assessment by professionals with training, so the relevant results should be interpreted more cautiously. Finally, although a subgroup analysis based on the details of OCBT intervention measures was conducted, there was still high heterogeneity between studies.

### 4.3. Relevance for Clinical Practice

In this study, we systematically searched, evaluated, and analyzed randomized controlled studies related to OBCT for postpartum depression using evidence-based medicine methods, focusing on the overall impact of OCBT on postpartum depression and summarizing the latest research evidence. At the same time, based on the intervention measures of OCBT and whether there was professional guidance, a relatively sufficient subgroup analysis was carried out to sort out the normative measures for the implementation of OCBT intervention; to provide more normative, practical, and efficient OCBT for women with postpartum depression; and to provide more scientific evidence for the clinical practice of OCBT. Finally, we conducted a preliminary discussion about the role and tasks of nurses in OCBT in order to promote nurses to participate in OCBT scientifically, professionally, and effectively, and give full play to the important role of nurses in the field of disease treatment, nursing, and rehabilitation. Future systematic reviews are encouraged to conduct subgroup analyses of more outcome indicators to determine the specific effects of different OCBT intervention measures on postpartum depression.

## 5. Conclusions

This systematic review and meta-analysis evaluated the effects of OCBT on postpartum depression. The results showed that OCBT was effective at improving postpartum depression symptoms. Furthermore, an OCBT intervention duration of nine weeks or longer and an intervention number of twelve or fewer significantly improved postpartum depression symptoms. In the implementation of OCBT, providing professional guidance could be more effective. However, the current evidence could not prove the effect of each intervention duration on postpartum depression. Finally, nurses could participate in OBCT interventions for postpartum depression to improve access to evidence-based treatment. Future studies should be encouraged to explore the full impact of OCBT on more relevant outcomes of postpartum depression.

## Figures and Tables

**Table 1:** The detailed characteristics of the eighteen included studies.

Author (Year)	Country	Participant	Sample Size (I/C)	Intervention								Comparison	Outcome	
				Total Duration	Number	Each Duration	Platform	Module Number	Form	Guidance Provided	Nurse Participation		Measurement	Data- Collection Time Point
Babiy et al., 2024 [24]	Canada	Postpartum women	405 (202/203)	12 weeks	1	6 h	Zoom	4	Team	Yes	No	TAU + WLC	EPDS	T0 T1
Carona et al., 2023 [25]	Portugal	Postpartum women	1053 (542/511)	8 weeks	5	45 min	Website	5	Personal	No	No	TAU	EPDS	T0 T1
Carona et al., 2023 [26]	Portugal	Postpartum women	1053 (542/511)	8 weeks	5	45 min	Website	5	Personal	No	No	TAU	EPDS	T0 T2
Fonseca et al., 2020 [27]	Portugal	Postpartum women	194 (98/96)	8 weeks	5	/	Website	5	Personal	No	No	WLC	EPDS	T0 T1
Huh et al., 2023 [17]	Canada	Postpartum women	159 (80/79)	9 weeks	9	2 h	Zoom	9	Team	Yes	Yes	TAU	EPDS	T0 T1 T2
Jannati et al., 2020 [28]	Iran	Postpartum women	78 (39/39)	8 weeks	8	45–60 min	APP	8	Personal	No	No	TAU	EPDS	T0 T1
Loughnan et al., 2019 [34]	Australia	Postpartum women	131 (69/62)	6 weeks	3	/	Website	3	Personal	No	No	TAU	EPDS	T0 T1 T2
Merza et al., 2023 [29]	Canada	Postpartum women	183 (92/91)	9 weeks	9	2 h	Zoom	9	Team	Yes	No	TAU + WLC	EPDS	T0 T1 T2
Milgrom et al., 2016 [35]	Australia	Postpartum women	43 (21/22)	12 weeks	6	/	Website	6	Personal	Yes	No	TAU	BDI	T0 T1
Milgrom et al., 2021 [21]	Australia	Postpartum women	77 (39/38)	9 weeks	6	/	Website	6	Personal	Yes	No	TAU	BDI	T0 T1 T2
Ngai et al., 2015 [18]	China	Postpartum women	397 (197/200)	5 weeks	5	20–30 min	APP	5	Personal	Yes	Yes	TAU	EPDS	T0 T1 T2
O’Mahen et al., 2013 [36]	UK	Postpartum women	910 (462/448)	15 weeks	11	Up to 40 min	Website	11	Personal	Yes	No	TAU	EPDS	T0 T1
O’Mahen et al., 2014 [37]	UK	Postpartum women	83 (41/42)	12 weeks	12	/	Website	12	Personal	Yes	No	TAU	EPDS	T0 T1 T2
Pugh et al., 2016 [38]	Canada	Postpartum women	50 (25/25)	10 weeks	7	/	Website	7	Personal	Yes	No	WLC	EPDS	T0 T1 T2
Seo et al., 2022 [22]	South Korea	Postpartum women	100 (50/50)	8 weeks	/	/	APP	/	Personal	/	No	TAU	EPDS	T0 T1 T2
Shariatpanahi et al., 2022 [30]	Iran	Postpartum women	102 (52/50)	16 weeks	16	/	Telegram	/	Personal	Yes	No	Medicine	EPDS	T0 T1
Van et al., 2021 [31]	Canada	Postpartum women	403 (202/201)	12 weeks	4	1 day	Zoom	4	Team	Yes	No	TAU	EPDS	T0 T1
Wozney et al., 2017 [39]	Canada	Postpartum women	62 (32/30)	12 weeks	12	/	Website	12	Personal	Yes	No	TAU	BDI	T0 T1 T2

Number: number of interventions; Module Number: number of modules set by OCBT (users could complete all modules in one intervention or one module in one intervention, which was different from Number); TAU: treatment as usual; WLC: waitlist control; EPDS: Edinburgh Postnatal Depression Scale; BDI: Beck Depression Inventory; T0: baseline; T1: post-intervention; T2: follow-up.

## Data Availability

The raw data for this study can be found in the article and the Supplementary Material; further inquiries can be directed to the corresponding author.

## Supplementary Materials

The following supporting information can be downloaded at https://www.mdpi.com/article/10.3390/healthcare13070696/s1. Table S1: PRISMA 2020 Checklist; Table S2: Specific search strategies and results of each database/register; Table S3: Summary of risk of bias assessment; Table S4: Summary of the results of sensitivity analyses; Table S5: Differences between protocol and review; Figure S1: Forest plot of the effect of OCBT on postpartum depression symptoms for different intervention durations; Figure S2: Forest plot of the effect of OCBT on postpartum depression symptoms for different intervention module numbers; Figure S3: Forest plot of the effect of OCBT on postpartum depression symptoms for different intervention forms; Figure S4: Summary of funnel plots; Figure S5: GRADE evidence profile; File S1: Studies ineligible following full-text review (N = 57); File S2: The titles of the included studies (N = 18); File S3: The complete process of contacting the original authors for clarification or data provision.

## References

[1] Mughal S., Azhar Y., Siddiqui W. (2023). Postpartum Depression.

[2] Wang Z., Liu J., Shuai H., Cai Z., Fu X., Liu Y., Xiao X., Zhang W., Krabbendam E., Liu S. (2021). Mapping global prevalence of depression among postpartum women. Transl. Psychiatry.

[3] Liu X., Wang S., Wang G. (2022). Prevalence and Risk Factors of Postpartum Depression in Women: A Systematic Review and Meta-analysis. J. Clin. Nurs..

[4] Nisar A., Yin J., Waqas A., Bai X., Wang D., Rahman A., Li X. (2020). Prevalence of perinatal depression and its determinants in Mainland China: A systematic review and meta-analysis. J. Affect. Disord..

[5] Saharoy R., Potdukhe A., Wanjari M., Taksande A.B. (2023). Postpartum Depression and Maternal Care: Exploring the Complex Effects on Mothers and Infants. Cureus.

[6] Dowse E., Chan S., Ebert L., Wynne O., Thomas S., Jones D., Fealy S., Evans T.-J. (2020). Impact of Perinatal Depression and Anxiety on Birth Outcomes: A Retrospective Data Analysis. Matern. Child Health J..

[7] Higgins N.E., Rose M.J., Gardner T.J., Crawford J.N. (2023). Perinatal Depression Treatment Guidelines for Obstetric Providers. Obstet. Gynecol. Clin. N. Am..

[8] Beck A., Rush A., Shaw B., Emery G. (1979). The Cognitive Therapy of Depression.

[9] Dryden W., Freeman A., Felgoise S.H., Nezu C.M., Nezu A.M., Reinecke M.A. (2010). Handbook of Cognitive-Behavioral Therapies.

[10] Dobson D.J.G., Dobson K.S. (2009). Evidence-Based Practice of Cognitive-Behavioral Therapy.

[11] National Institute for Health and Care Excellence (2018). Antenatal and Postnatal Mental Health—Clinical Management and Service Guidance (Updated ed.).

[12] Gelenberg A.J., Freeman M.P., Markowitz J.C., Rosenbaum J.F., Thase M.E., Trivedi M.H. (2010). Practice Guideline for the Treatment of Patients with Major Depressive Disorder.

[13] British Thoracic Society (2023). Scottish Intercollegiate Guidelines Network A National Clinical Guideline: Perinatal Mental Health Conditions.

[14] Amani B., Merza D., Savoy C., Streiner D., Bieling P., Ferro M.A., Van Lieshout R.J. (2021). Peer-Delivered Cognitive-Behavioral Therapy for Postpartum Depression: A Randomized Controlled Trial. J. Clin. Psychiatry.

[15] Ammerman R.T., Putnam F.W., Altaye M., Stevens J., Teeters A.R., Van Ginkel J.B. (2013). A clinical trial of in-home CBT for depressed mothers in home visitation. Behav. Ther..

[16] Milgrom J., Holt C.J., Gemmill A.W., Ericksen J., Leigh B., Buist A., Schembri C. (2011). Treating postnatal depressive symptoms in primary care: A randomised controlled trial of GP management, with and without adjunctive counselling. BMC Psychiatry.

[17] Huh K., Layton H., Savoy C.D., Ferro M.A., Bieling P.J., Hicks A., Van Lieshout R.J. (2023). Online Public Health Nurse–Delivered Group Cognitive Behavioral Therapy for Postpartum Depression: A Randomized Controlled Trial During the COVID-19 Pandemic. J. Clin. Psychiatry.

[18] Ngai F.-W., Wong P.W.-C., Leung K.-Y., Chau P.-H., Chung K.-F. (2015). The Effect of Telephone-Based Cognitive-Behavioral Therapy on Postnatal Depression: A Randomized Controlled Trial. Psychother. Psychosom..

[19] Denecke K., Schmid N., Nüssli S. (2022). Implementation of Cognitive Behavioral Therapy in e-Mental Health Apps: Literature Review. J. Med. Internet Res..

[20] Sheeber L.B., Seeley J.R., Feil E.G., Davis B., Sorensen E., Kosty D.B., Lewinsohn P.M. (2012). Development and pilot evaluation of an Internet-facilitated cognitive-behavioral intervention for maternal depression. J. Consult. Clin. Psychol..

[21] Milgrom J., Danaher B.G., Seeley J.R., Holt C.J., Holt C., Ericksen J., Tyler M.S., Gau J.M., Gemmill A.W. (2021). Internet and Face-to-face Cognitive Behavioral Therapy for Postnatal Depression Compared with Treatment as Usual: Randomized Controlled Trial of MumMoodBooster. J. Med. Internet Res..

[22] Seo J.-M., Kim S.-J., Na H., Kim J.-H., Lee H. (2022). Effectiveness of a Mobile Application for Postpartum Depression Self-Management: Evidence from a Randomised Controlled Trial in South Korea. Healthcare.

[23] Roman M., Constantin T., Bostan C.M. (2020). The efficiency of online cognitive-behavioral therapy for postpartum depressive symptomatology: A systematic review and meta-analysis. Women Health.

[24] Babiy Z., Layton H., Savoy C.D., Xie F., Brown J.S.L., Bieling P.J., Streiner D.L., Ferro M.A., Van Lieshout R.J. (2024). One-Day Peer-Delivered Cognitive Behavioral Therapy-Based Workshops for Postpartum Depression: A Randomized Controlled Trial. Psychother. Psychosom..

[25] Carona C., Pereira M., Araújo-Pedrosa A., Canavarro M.C., Fonseca A. (2023). The Efficacy of Be a Mom, a Web-Based Intervention to Prevent Postpartum Depression: Examining Mechanisms of Change in a Randomized Controlled Trial. JMIR Ment. Health.

[26] Carona C., Pereira M., Araújo-Pedrosa A., Monteiro F., Cristina Canavarro M., Fonseca A. (2023). For Whom and for How Long Does the “Be a Mom” Intervention Work? A Secondary Analysis of Data from a Randomized Controlled Trial Exploring the Mid-Term Efficacy and Moderators of Treatment Response. Behav. Ther..

[27] Fonseca A., Alves S., Monteiro F., Gorayeb R., Canavarro M.C. (2020). Be a mom, a Web-Based Intervention to Prevent Postpartum Depression: Results From a Pilot Randomized Controlled Trial. Behav. Ther..

[28] Jannati N., Mazhari S., Ahmadian L., Mirzaee M. (2020). Effectiveness of an app-based cognitive behavioral therapy program for postpartum depression in primary care: A randomized controlled trial. Int. J. Med. Inf..

[29] Merza D., Amani B., Savoy C., Babiy Z., Bieling P.J., Streiner D.L., Ferro M.A., Van Lieshout R.J. (2023). Online peer-delivered group cognitive-behavioral therapy for postpartum depression: A randomized controlled trial. Acta Psychiatr. Scand..

[30] Shariatpanahi G., Effatpanah M., Moienafshar A., Shariati M., Kheiltash A., Ahadpourkhanghah E., Khaneghah A.A. (2023). Comparing the Effectiveness of Internet-Based Cognitive Behavioral Therapy and Drug Therapy for Treating Postpartum Depression and Children Weight Gain: A Randomized Clinical Trial. Int. J. High Risk Behav. Addict..

[31] Van Lieshout R.J., Layton H., Savoy C.D., Brown J.S.L., Ferro M.A., Streiner D.L., Bieling P.J., Feller A., Hanna S. (2021). Effect of Online 1-Day Cognitive Behavioral Therapy–Based Workshops Plus Usual Care vs Usual Care Alone for Postpartum Depression: A Randomized Clinical Trial. JAMA Psychiatry.

[32] Higgins J., Thomas J., Chandler J., Cumpston M., Li T., Page M. Cochrane Handbook for Systematic Reviews of Interventions Version 6.3 (Updated February 2022). https://training.cochrane.org/handbook/current.

[33] Page M.J., McKenzie J.E., Bossuyt P.M., Boutron I., Hoffmann T.C., Mulrow C.D., Shamseer L., Tetzlaff J.M., Akl E.A., Brennan S.E. (2021). The PRISMA 2020 statement: An updated guideline for reporting systematic reviews. BMJ.

[34] Loughnan S.A., Butler C., Sie A.A., Grierson A.B., Chen A.Z., Hobbs M.J., Joubert A.E., Haskelberg H., Mahoney A., Holt C. (2019). A randomised controlled trial of ‘MUMentum postnatal’: Internet-delivered cognitive behavioural therapy for anxiety and depression in postpartum women. Behav. Res. Ther..

[35] Milgrom J., Danaher B.G., Gemmill A.W., Holt C., Holt C.J., Seeley J.R., Tyler M.S., Ross J., Ericksen J. (2016). Internet Cognitive Behavioral Therapy for Women With Postnatal Depression: A Randomized Controlled Trial of MumMoodBooster. J. Med. Internet Res..

[36] O’Mahen H.A., Woodford J., McGinley J., Warren F.C., A Richards D., Lynch T.R., Taylor R.S. (2013). Internet-based behavioral activation—Treatment for postnatal depression (Netmums): A randomized controlled trial. J. Affect. Disord..

[37] O’Mahen H.A., Richards D.A., Woodford J., Wilkinson E., McGinley J., Taylor R.S., Warren F.C. (2014). Netmums: A phase II randomized controlled trial of a guided Internet behavioural activation treatment for postpartum depression. Psychol. Med..

[38] Pugh N.E., Hadjistavropoulos H.D., Dirkse D. (2016). A Randomised Controlled Trial of Therapist-Assisted, Internet-Delivered Cognitive Behavior Therapy for Women with Maternal Depression. PLoS ONE.

[39] Wozney L., Olthuis J., Lingley-Pottie P., McGrath P.J., Chaplin W., Elgar F., Cheney B., Huguet A., Turner K., Kennedy J. (2017). Strongest Families^TM^ Managing Our Mood (MOM): A randomized controlled trial of a distance intervention for women with postpartum depression. Arch. Womens Ment. Health.

[40] Buss J.F., Steinberg J.S., Banks G., Horani D., Rutter L.A., Wasil A.R., Ramirez I., Lorenzo-Luaces L. (2024). Availability of Internet-Based Cognitive-Behavioral Therapies for Depression: A Systematic Review. Behav. Ther..

[41] Wilhelm S., Weingarden H., Ladis I., Braddick V., Shin J., Jacobson N.C. (2020). Cognitive-Behavioral Therapy in the Digital Age: Presidential Address. Behav. Ther..

[42] Pettman D., O’Mahen H., Blomberg O., Svanberg A.S., Von Essen L., Woodford J. (2023). Effectiveness of cognitive behavioural therapy-based interventions for maternal perinatal depression: A systematic review and meta-analysis. BMC Psychiatry.

[43] Udd-Granat L., Lahti J., Donnelly M., Treanor C., Pirkola S.P., Lallukka T., Kouvonen A. (2023). Internet-delivered cognitive behavioral therapy (iCBT) for common mental disorders and subsequent sickness absence: A systematic review and meta-analysis. Scand. J. Public Health.

[44] Qiao J., Wang Y., Li X., Jiang F., Zhang Y., Ma J., Song Y., Ma J., Fu W., Pang R. (2021). A Lancet Commission on 70 years of women’s reproductive, maternal, newborn, child, and adolescent health in China. Lancet.

[45] (2023). The Lancet Perinatal depression: A neglected aspect of maternal health. Lancet.

[46] Bai X., Song Z., Zhou Y., Wang X., Wang Y., Zhang D. (2021). Bibliometrics and Visual Analysis of the Research Status and Trends of Postpartum Depression From 2000 to 2020. Front. Psychol..

[47] Dosani A., Arora H., Mazmudar S. (2020). mHealth and Perinatal Depression in Low-and Middle-Income Countries: A Scoping Review of the Literature. Int. J. Environ. Res. Public Health.

[48] Raaj S., Verghese V., Tharmaseelan M., Duffy R., Sinnadorai N.K.S.T.N.K. (2023). Perinatal mental health in Malaysia: Understanding the treatment gap and recommendations for the future. BJPsych Int..

[49] National Institute for Health and Care Excellence Depression in Adults: Treatment and Management. https://www.nice.org.uk/guidance/ng222.

[50] Cuijpers P., Franco P., Ciharova M., Miguel C., Segre L., Quero S., Karyotaki E. (2023). Psychological treatment of perinatal depression: A meta-analysis. Psychol. Med..

[51] Dear B.F., Titov N., Schwencke G., Andrews G., Johnston L., Craske M.G., McEvoy P. (2011). An open trial of a brief transdiagnostic internet treatment for anxiety and depression. Behav. Res. Ther..

[52] Lau Y., Htun T.P., Wong S.N., Tam W.S.W., Klainin-Yobas P. (2017). Therapist-Supported Internet-Based Cognitive Behavior Therapy for Stress, Anxiety, and Depressive Symptoms Among Postpartum Women: A Systematic Review and Meta-Analysis. J. Med. Internet Res..

[53] Morgan C., Mason E., Newby J.M., Mahoney A.E., Hobbs M.J., McAloon J., Andrews G. (2017). The effectiveness of unguided internet cognitive behavioural therapy for mixed anxiety and depression. Internet Interv..

[54] Dear B.F., Fogliati V.J., Fogliati R., Johnson B., Boyle O., Karin E., Gandy M., Kayrouz R., Staples L.G., Titov N. (2018). Treating anxiety and depression in young adults: A randomised controlled trial comparing clinician-guided versus self-guided Internet-delivered cognitive behavioural therapy. Aust. N. Z. J. Psychiatry.

[55] Laurant M., van der Biezen M., Wijers N., Watananirun K., Kontopantelis E., van Vught A.J. (2018). Nurses as substitutes for doctors in primary care. Cochrane Database Syst. Rev..

[56] Hong S., Lee S., Song K., Kim M., Kim Y., Kim H., Kim H. (2023). A nurse-led mHealth intervention to alleviate depressive symptoms in older adults living alone in the community: A quasi-experimental study. Int. J. Nurs. Stud..

