# Supplementary material for: The Effects of Online Cognitive Behavioral Therapy on Postpartum Depression: A Systematic Review and Meta-Analysis
Source: Healthcare (Basel). 2025 Mar 21;13(7):696. doi: 10.3390/healthcare13070696 (PMC11989130; doi:10.3390/healthcare13070696)
Supplement: Supplementary file 1 [file healthcare-13-00696-s001.zip › healthcare-3404864-supplementary.pdf]

Supplementary Material

**Supplementary Table S1**	PRISMA 2020 Checklist
**Supplementary Table S2**	Specific search strategies and results of each database/register
**Supplementary Table S3**	Summary of risk of bias assessment
**Supplementary Table S4**	Summary of the results of sensitivity analyses
**Supplementary Table S5**	Differences between protocol and review
**Supplementary Figure S1**	Forest plot of the effect of OCBT on postpartum depression symptom in different each intervention duration
**Supplementary Figure S2**	Forest plot of the effect of OCBT on postpartum depression symptom in different intervention module number
**Supplementary Figure S3**	Forest plot of the effect of OCBT on postpartum depression symptom in different intervention form
**Supplementary Figure S4**	Summary of funnel plots
**Supplementary Figure S5**	GRADE evidence profile
**Supplementary File S1**	Studies ineligible following full-text review (N=57)
**Supplementary File S2**	The titles of the included studies (N=18)
**Supplementary File S3**	The complete process of contacting the original authors for clarification or data provision

Supplementary Table S1 *PRISMA2020* Checklist

Section and Topic	Item	Checklist item	Location where item is reported
**TITLE**			
Title	1	Identify the report as a systematic review.	Title
**ABSTRACT**			
Abstract	2	See the PRISMA 2020 for Abstracts checklist.	Abstract
**INTRODUCTION**			
Rationale	3	Describe the rationale for the review in the context of existing knowledge.	1. Introduction
Objectives	4	Provide an explicit statement of the objective(s) or question(s) the review addresses.	1. Introduction
**METHODS**			
Eligibility criteria	5	Specify the inclusion and exclusion criteria for the review and how studies were grouped for the syntheses.	2.2. Eligibility criteria
Information sources	6	Specify all databases, registers, websites, organisations, reference lists and other sources searched or consulted to identify studies. Specify the date when each source was last searched or consulted.	2.1. Search strategy
Search strategy	7	Present the full search strategies for all databases, registers and websites, including any filters and limits used.	2.1. Search strategy & Supplementary Table S2
Selection process	8	Specify the methods used to decide whether a study met the inclusion criteria of the review, including how many reviewers screened each record and each report retrieved, whether they worked independently, and if applicable, details of automation tools used in the process.	2.3. Study selection
Data collection process	9	Specify the methods used to collect data from reports, including how many reviewers collected data from each report, whether they worked independently, any processes for obtaining or confirming data from study investigators, and if applicable, details of automation tools used in the process.	2.5. Data extraction
Data items	10a	List and define all outcomes for which data were sought. Specify whether all results that were compatible with each outcome domain in each study were sought (e.g. for all measures, time points, analyses), and if not, the methods used to decide which results to collect.	2.5. Data extraction & Table 1
	10b	List and define all other variables for which data were sought (e.g. participant and intervention characteristics, funding sources). Describe any assumptions made about any missing or unclear information.	2.5. Data extraction & Table 1
Study risk of bias assessment	11	Specify the methods used to assess risk of bias in the included studies, including details of the tool(s) used, how many reviewers assessed each study and whether they worked independently, and if applicable, details of automation tools used in the process.	2.4. Quality assessment
Effect measures	12	Specify for each outcome the effect measure(s) (e.g. risk ratio, mean difference) used in the synthesis or presentation of results.	2.6. Data analyses
Synthesis methods	13a	Describe the processes used to decide which studies were eligible for each synthesis (e.g. tabulating the study intervention characteristics and comparing against the planned groups for each synthesis (item #5)).	2.6. Data analyses
	13b	Describe any methods required to prepare the data for presentation or synthesis, such as handling of missing summary statistics, or data conversions.	2.6. Data analyses
	13c	Describe any methods used to tabulate or visually display results of individual studies and syntheses.	2.6. Data analyses
	13d	Describe any methods used to synthesize results and provide a rationale for the choice(s). If meta-analysis was performed, describe the model(s), method(s) to identify the presence and extent of statistical heterogeneity, and software package(s) used.	2.6. Data analyses
	13e	Describe any methods used to explore possible causes of heterogeneity among study results (e.g. subgroup analysis, meta-regression).	2.6. Data analyses
	13f	Describe any sensitivity analyses conducted to assess robustness of the synthesized results.	2.6. Data analyses
Reporting bias assessment	14	Describe any methods used to assess risk of bias due to missing results in a synthesis (arising from reporting biases).	2.6. Data analyses
Certainty assessment	15	Describe any methods used to assess certainty (or confidence) in the body of evidence for an outcome.	2.7. Certainty assessment of the body of

Section and Topic	Item	Checklist item	Location where item is reported
			evidence
**RESULTS**			
Study selection	16a	Describe the results of the search and selection process, from the number of records identified in the search to the number of studies included in the review, ideally using a flow diagram.	3.1. Search results and study selection & Figure 1
	16b	Cite studies that might appear to meet the inclusion criteria, but which were excluded, and explain why they were excluded.	3.1. Search results and study selection & Supplementary File S1
Study characteristics	17	Cite each included study and present its characteristics.	3.2. Study characteristics & Table 1
Risk of bias in studies	18	Present assessments of risk of bias for each included study.	3.3. Risk of bias assessment & Figure 2 & Supplementary Table S3
Results of individual studies	19	For all outcomes, present, for each study: (a) summary statistics for each group (where appropriate) and (b) an effect estimate and its precision (e.g. confidence/credible interval), ideally using structured tables or plots.	3.4. Effects of Online Cognitive Behavioral Therapy on postpartum depression outcomes & Figures 3-8
Results of syntheses	20a	For each synthesis, briefly summarise the characteristics and risk of bias among contributing studies.	3.4. Effects of Online Cognitive Behavioral Therapy on postpartum depression outcomes
	20b	Present results of all statistical syntheses conducted. If meta-analysis was done, present for each the summary estimate and its precision (e.g. confidence/credible interval) and measures of statistical heterogeneity. If comparing groups, describe the direction of the effect.	3.4. Effects of Online Cognitive Behavioral Therapy on postpartum depression outcomes & Figures 3-8
	20c	Present results of all investigations of possible causes of heterogeneity among study results.	3.4. Effects of Online Cognitive Behavioral Therapy on postpartum depression outcomes
	20d	Present results of all sensitivity analyses conducted to assess the robustness of the synthesized results.	3.5. Sensitivity analyses and assessment of publication bias & Supplementary Table S4
Reporting biases	21	Present assessments of risk of bias due to missing results (arising from reporting biases) for each synthesis assessed.	3.5. Sensitivity analyses and assessment of publication bias & Supplementary Figure S4
Certainty of evidence	22	Present assessments of certainty (or confidence) in the body of evidence for each outcome assessed.	3.6. Certainty of the body of evidence & Supplementary Figure S5
**DISCUSSION**			
Discussion	23a	Provide a general interpretation of the results in the context of other evidence.	4.1. Interpretation of findings
	23b	Discuss any limitations of the evidence included in the review.	4.2. Limitations
	23c	Discuss any limitations of the review processes used.	4.2. Limitations
	23d	Discuss implications of the results for practice, policy, and future research.	4.3. Relevance for clinical practice
**OTHER INFORMATION**			
Registration and protocol	24a	Provide registration information for the review, including register name and registration number, or state that the review was not registered.	2. Materials and Methods
	24b	Indicate where the review protocol can be accessed, or state that a protocol was not prepared.	2. Materials and Methods
	24c	Describe and explain any amendments to information provided at registration or in the protocol.	Supplementary Table S5
Support	25	Describe sources of financial or non-financial support for the review, and the role of the funders or sponsors in the review.	Funding
Competing interests	26	Declare any competing interests of review authors.	Conflicts of Interest
Availability of data, code and other materials	27	Report which of the following are publicly available and where they can be found: template data collection forms; data extracted from included studies; data used for all analyses; analytic code; any other materials used in the review.	Supplementary materials

From: Page MJ, McKenzie JE, Bossuyt PM, Boutron I, Hoffmann TC, Mulrow CD, et al. The PRISMA 2020 statement: an updated guideline for reporting systematic reviews. BMJ 2021;372:n71. doi: 10.1136/bmj.n71

Supplementary Table S2 Specific search strategies and results of each database/register

Search date: from inception to December 31, 2023

Database/Register	Search strategies	Results
PubMed	#1 "Depression, Postpartum"[Mesh]	7,784
	#2 "postnatal depress*" OR "postpartum depress*" OR "puerperal depress*" OR "post-partum depress*" OR "post-natal depress*" OR "maternal depress*"	15,454
	#3 #1 OR #2	15,454
	#4 "Cognitive Behavioral Therapy" [Mesh]	37,871
	#5 "cognitive behavior* therapy"[All Fields] OR "cognitive behaviour* therapy"[All Fields] OR "cognitive behavior* treatment"[All Fields] OR "cognitive behaviour* treatment"[All Fields] OR "cognitive behavior* intervention"[All Fields] OR "cognitive behaviour* intervention"[All Fields] OR "cognitive therapy"[All Fields] OR "cognitive treatment"[All Fields] OR "cognitive intervention"[All Fields] OR "cognition therapy"[All Fields] OR "cognition treatment"[All Fields] OR "cognition intervention"[All Fields] OR "behavior* therapy"[All Fields] OR "behaviour* therapy"[All Fields] OR "behavior* treatment"[All Fields] OR "behaviour* treatment"[All Fields] OR "behavior* intervention"[All Fields] OR "behaviour* intervention"[All Fields]	46,596
	#6 "cognitive reorganization"[All Fields] OR "cognitive restructuring"[All Fields] OR "cognitive reconstruction"[All Fields]	1,178
	#7 "behavior* activation"[All Fields] OR "behaviour* activation"[All Fields] OR "behavior* correction"[All Fields] OR "behaviour* correction"[All Fields] OR "behavior* modification"[All Fields] OR "behaviour* modification"[All Fields] OR "behavior* remedy"[All Fields] OR "behavior* adjustment"[All Fields] OR "behaviour* adjustment"[All Fields]	4,770
	#8 #4 OR #5 OR #6 OR #7	79,649
	#9 "online" [All Fields] OR "digital" [All Fields] OR "electronic" [All Fields] OR "web" [All Fields] OR "website" [All Fields] OR "internet" [All Fields] OR "computer" [All Fields] OR "mobile application*" [All Fields] OR "telephone" [All Fields] OR "internet-based" [All Fields] OR "internet-delivered" [All Fields] OR "web-based" [All Fields] OR "computerized" [All Fields] OR "computer-assisted" [All Fields]	4,414,076
	#10 #8 AND #9	17,505
	#11 ("ICBT" [All Fields] OR "CCBT" [All Fields] OR "OCBT" [All Fields]) NOT ("intracavitary brachytherapy"[All Fields] OR "clear cell borderline tumor"[All Fields] OR "carotid body tumor"[All Fields] OR "chemo-brachytherapy"[All Fields] OR "capacity building tools"[All Fields] OR "haplotype"[All Fields])	1,264
	#12 #10 OR #11	17,945
	#13 #3 AND #12	148
Embase	#1 'postnatal depression'/exp OR 'postnatal depression'	19,456
	#2 'postnatal depress*' OR 'postpartum depress*' OR 'post-partum depress*' OR 'post-natal depress*' OR 'maternal depress*' OR 'puerperal depress*'	23,006
	#3 #1 OR #2	23,006
	#4 'cognitive behavioral therapy'/exp OR 'cognitive behavioral therapy' OR 'cognitive behavioral stress management' OR 'cognitive processing therapy' OR 'cognitive restructuring' OR 'dialectical behavior therapy' OR 'mindfulness-based cognitive therapy' OR 'mindfulness-based stress reduction' OR 'problem solving therapy'	44,821
	#5 'cognitive behavio* therap*' OR 'cognitive behavio* treatment' OR 'cognitive behavio* intervention' OR 'cognitive therap*' OR 'cognitive treatment' OR 'cognitive intervention' OR 'cognition therap*' OR 'cognition treatment' OR 'cognition intervention' OR 'behavio* therap*' OR 'behavio* treatment' OR 'behavio* intervention'	133,005
	#6 'cogniti* recombination' OR 'cogniti* regroup' OR 'cogniti* recombine' OR 'cogniti* reorganization' OR 'cogniti* restructuring' OR 'cogniti* reconstruction' OR 'behavio* activat*' OR 'behavio* correct' OR 'behavio* correction' OR 'behavio* rectification' OR 'behavio* reclamation' OR 'behavio* modification' OR 'behavio* remedy' OR 'behavio* adjustment'	20,982
	#7 #4 OR #5 OR #6	151,668
	#8 'online' OR 'digital' OR 'electronic' OR 'web' OR 'website' OR 'internet' OR 'computer' OR 'mobile application*' OR 'telephone' OR 'internet-based' OR 'internet-delivered' OR 'web-based' OR 'computerized' OR 'computer-assisted'	18,546,547
	#9 #7 AND #8	83,935
	#10 'ICBT' OR 'CCBT' OR 'OCBT'	1,893
	#11 #9 OR #10	84,594
	#12 #3 AND #11	789
CINAHL Plus	#1 MH "Depression, Postpartum"	7,116
	#2 SU "postnatal depress*" OR SU "postpartum depress*" OR SU "puerperal depress*" OR SU "post-partum depress*" OR SU "post-natal depress*" OR SU "maternal depress*"	3,019
	#3 #1 OR #2	8,453
	#4 MH "Cognitive Therapy" OR MH "Behavior Therapy" OR MH "Cognitive Restructuring" OR MH "Cognitive Remediation" OR MH "Behavior Modification"	37,133
	#5 TX "cognitive behavioral therapy" OR TX "cogniti* behavio* therap*" OR TX "cogniti* behavio* treatment" OR TX "cogniti* behavio* intervention" OR	53,268

	SU "cogniti* therap*" OR SU "cogniti* treatment" OR SU "cogniti* intervention" OR SU "behavio* therap*" OR SU "behavio* treatment" OR SU "behavio* intervention"	
	#6 SU "cogniti* recombination" OR SU "cogniti* regroup" OR SU "cogniti* recombine" OR SU "cogniti* reorganization" OR SU "cogniti* restructuring" OR SU "cogniti* reconstruction"	114
	#7 SU "behavio* activat*" OR SU "behavio* correct" OR SU "behavio* correction" OR SU "behavio* rectification" OR SU "behavio* reclamation" OR SU "behavio* modification" OR SU "behavio* remedy" OR SU "behavio* adjustment"	2,673
	#8 #4 OR #5 OR #6 OR #7	55,838
	#9 SU online OR SU digital OR SU electronic OR SU web OR SU website OR SU internet OR SU computer OR SU mobile application* OR SU telephone OR SU internet-based OR SU internet-delivered OR SU web-based OR SU computerized OR SU computer-assisted	364,465
	#10 #8 AND #9	3,921
	#11 TX ICBT OR TX CCBT OR TX OCBT	673
	#12 #10 OR #11	4,258
	#13 #3 AND #12	44
Web of Science	#1 TS=("postnatal depress*" OR "postpartum depress*" OR "puerperal depress*" OR "post-partum depress*" OR "post-natal depress*" OR "maternal depress*")	21,882
	#2 TS=("cognitive behavioral therapy" OR "cogniti* behavio* therap*" OR "cogniti* behavio* treatment" OR "cogniti* behavio* intervention" OR "cogniti* therap*" OR "cogniti* treatment" OR "cogniti* intervention" OR "behavio* therap*" OR "behavio* treatment" OR "behavio* intervention")	79,161
	#3 TS=("cogniti* recombination" OR "cogniti* regroup" OR "cogniti* recombine" OR "cogniti* reorganization" OR "cogniti* restructuring" OR "cogniti* reconstruction")	1,556
	#4 TS=("behavio* activat*" OR "behavio* correct" OR "behavio* correction" OR "behavio* rectification" OR "behavio* reclamation" OR "behavio* modification" OR "behavio* remedy" OR "behavio* adjustment")	10,760
	#5 #2 OR #3 OR #4	89,584
	#6 TS=("online" OR "digital" OR "electronic" OR "web" OR "website" OR "internet" OR "computer" OR "mobile application" OR "telephone" OR "internet-based" OR "internet-delivered" OR "web-based" OR "computerized" OR "computer-assisted")	4,074,665
	#7 #5 AND #6	14,059
	#8 TS=("ICBT" OR "CCBT" OR "OCBT")	1,498
	#9 #7 OR #8	14,508
	#10 #1 AND #9	229
The Cochrane Library	#1 MeSH descriptor: [Depression, Postpartum] explode all trees	979
	#2 ((postnatal NEXT depress*) OR (postpartum NEXT depress*) OR (puerperal NEXT depress*) OR (maternal NEXT depress*) OR (post-partum NEXT depress*) OR (post-natal NEXT depress*)):ti,ab,kw	2,975
	#3 #1 OR #2	3,135
	#4 MeSH descriptor: [Cognitive Behavioral Therapy] explode all trees	13,923
	#5 ((cognitive behavior* NEXT therapy) OR (cognitive behaviour* NEXT therapy) OR (cognitive behavior* NEXT treatment) OR (cognitive behaviour* NEXT treatment) OR (cognitive behavior* NEXT intervention) OR (cognitive behaviour* NEXT intervention) OR ("cognitive therapy") OR ("cognitive treatment") OR ("cognitive intervention") OR (behavior* NEXT therapy) OR (behaviour* NEXT therapy) OR (behavior* NEXT treatment) OR (behaviour* NEXT treatment) OR (behavior* NEXT intervention) OR (behaviour* NEXT intervention)):ti,ab,kw	41,087
	#6 (("cognitive reorganization") OR ("cognitive restructuring") OR ("cognitive reconstruction") OR ("cognitive recombination") OR ("cognitive regroup") OR ("cognitive recombine")):ti,ab,kw	992
	#7 ((behavior* NEXT activation) OR (behaviour* NEXT activation) OR (behavior* NEXT correct) OR (behaviour* NEXT correct) OR (behavior* NEXT correction) OR (behaviour* NEXT correction) OR (behavior* NEXT modification) OR (behaviour* NEXT modification) OR (behavior* NEXT remedy) OR (behaviour* NEXT remedy) OR (behavior* NEXT adjustment) OR (behaviour* NEXT adjustment) OR (behavior* NEXT rectification) OR (behaviour* NEXT rectification) OR (behavior* NEXT reclamation) OR (behaviour* NEXT reclamation)):ti,ab,kw	3,283
	#8 #4 OR #5 OR #6 OR #7	45,510
	#9 ((online) OR (digital) OR (electronic) OR (web) OR (website) OR (internet) OR (computer) OR (mobile NEXT application*) OR (telephone) OR (internet-based) OR (internet-delivered) OR (web-based) OR (computerized) OR (computer-assisted)):ti,ab,kw	190,999
	#10 #8 AND #9	11,137
	#11 ((ICBT) OR (CCBT) OR (OCBT)):ti,ab,kw	1,051
	#12 #10 OR #11	11,319
	#13 #3 AND #12	172
PsycINFO	#1 DE "Postpartum Depression"	6,367
	#2 SU "postnatal depress*" OR SU "postpartum depress*" OR SU "puerperal depress*" OR SU "post-partum depress*" OR SU "post-natal depress*" OR SU "maternal depress*"	8,281
	#3 #1 OR #2	8,281

	#4 DE "Cognitive Behavior Therapy" OR DE "Cognitive Therapy" OR DE "Behavior Therapy" OR DE "Cognitive Techniques" OR DE "Cognitive Psychology" OR DE "Cognitive Restructuring" OR DE "Cognitive Rehabilitation" OR DE "Cognitive Remediation" OR DE "Behavior Modification" OR DE "Behavior Contracting" OR DE "Mindfulness-Based Cognitive Therapy" OR DE "Trauma-Focused Cognitive Behavior Therapy" OR DE "Dialectical Behavior Therapy" OR DE "Classroom Behavior Modification"	93,273
	#5 SU "cognitive behavioral therapy" OR SU "cogniti* behavio* therap*" OR SU "cogniti* behavio* treatment" OR SU "cogniti* behavio* intervention" OR SU "cogniti* therap*" OR SU "cogniti* treatment" OR SU "cogniti* intervention" OR SU "behavio* therap*" OR SU "behavio* treatment" OR SU "behavio* intervention"	77,142
	#6 SU "cogniti* recombination" OR SU "cogniti* regroup" OR SU "cogniti* recombine" OR SU "cogniti* reorganization" OR SU "cogniti* restructuring" OR SU "cogniti* reconstruction"	1,175
	#7 SU "behavio* activat*" OR SU "behavio* correct" OR SU "behavio* correction" OR SU "behavio* rectification" OR SU "behavio* reclamation" OR SU "behavio* modification" OR SU "behavio* remedy" OR SU "behavio* adjustment"	15,953
	#8 #4 OR #5 OR #6 OR #7	100,414
	#9 SU online OR SU digital OR SU electronic OR SU web OR SU website OR SU internet OR SU computer OR SU mobile application* OR SU telephone OR SU internet-based OR SU internet-delivered OR SU web-based OR SU computerized OR SU computer-assisted	240,690
	#10 #8 AND #9	5,302
	#11 TX ICBT OR TX CCBT OR TX OCBT	830
	#12 #10 OR #11	5,483
	#13 #3 AND #12	37
China National Knowledge Infrastructure (CNKI)	#1 (SU = “产后抑郁”)	4,794
	#2 (SU = (“认知” + “行为” + “认知行为” + “CBT” + “认知重组” + “行为激活” + “行为矫正”))	2,062,735
	#3 (SU = (“网络” + “计算机” + “远程” + “互联网” + “在线” + “平台” + “系统” + “软件” + “程序” + “数字” + “邮件” + “移动” + “线上” + “APP” + “online”))	16,613,208
	#4 #1 AND #2 AND #3	78
WanFang Data Knowledge Service Platform (WanFang Data)	#1 题名或关键词:(“产后抑郁”)	7,864
	#2 题名或关键词:(“认知”) OR 题名或关键词:(“行为”) OR 题名或关键词:(“认知行为”) OR 题名或关键词:(“CBT”) OR 题名或关键词:(“认知重组”) OR 题名或关键词:(“行为激活”) OR 题名或关键词:(“行为矫正”)	1,093,578
	#3 题名或关键词:(“网络”) OR 题名或关键词:(“计算机”) OR 题名或关键词:(“远程”) OR 题名或关键词:(“互联网”) OR 题名或关键词:(“在线”) OR 题名或关键词:(“平台”) OR 题名或关键词:(“系统”) OR 题名或关键词:(“软件”) OR 题名或关键词:(“程序”) OR题名或关键词:(“数字”) OR 题名或关键词:(“邮件”) OR 题名或关键词:(“移动”) OR 题名或关键词:(“线上”) OR 题名或关键词:(“APP”) OR 题名或关键词:(“online”)	7,625,015
	#4 #1 AND #2 AND #3	22
China Science and Technology Journal Database (CQVIP)	#1 M=“产后抑郁”	5,741
	#2 M="认知" OR "行为" OR "认知行为" OR "CBT" OR "认知重组" OR "行为激活" OR "行为矫正"	899.927
	#3 M="网络" OR "计算机" OR "远程" OR "互联网" OR "在线" OR "平台" OR "系统" OR "软件" OR "程序"OR "数字" OR "邮件" OR "移动" OR "线上" OR "APP" OR "online"	6,251,169
	#4 #1 AND #2 AND #3	19
Chinese Biomedical Literature Service System (SinoMed)	#1 "抑郁症，产后"[不加权:扩展] OR "产后抑郁"[常用字段:智能]	6,630
	#2 "认知行为疗法"[不加权:扩展] OR "行为疗法"[不加权:扩展] OR "认知行为疗法"[常用字段:智能] OR "认知疗法"[常用字段:智能] OR "认知性心理疗法"[常用字段:智能] OR "正念"[常用字段:智能] OR "接受与投入疗法"[常用字段:智能] OR "认知行为"[常用字段:智能] OR "认知治疗"[常用字段:智能] OR "行为疗法"[常用字段:智能] OR "行为治疗"[常用字段:智能] OR "CBT"[常用字段:智能] OR "认知干预"[常用字段:智能] OR "行为干预"[常用字段:智能] OR "认知策略"[常用字段:智能] OR "行为策略"[常用字段:智能] OR "认知重组"[常用字段:智能] OR "行为激活"[常用字段:智能] OR "行为矫正"[常用字段:智能]	33,287
	#3 "计算机"[不加权:扩展] OR "远程医学"[不加权:扩展] OR "互联网干预"[不加权:扩展] OR "联机系统"[不加权:扩展] OR "计算机通信网络"[不加权:扩展] OR "信息系统"[不加权:扩展] OR "电子邮件"[不加权:扩展] OR "软件"[不加权:扩展] OR "数字技术"[不加权:扩展] OR "网络"[常用字段:智能] OR "计算机"[常用字段:智能] OR "远程"[常用字段:智能] OR "互联网"[常用字段:智能] OR "在线"[常用字段:智能] OR "平台"[常用字段:智能] OR "系统"[常用字段:智能] OR "软件"[常用字段:智能] OR "程序"[常用字段:智能] OR "APP"[常用字段:智能] OR "邮件"[常用字段:智能] OR "移动"[常用字段:智能] OR "线上"[常用字段:智能] OR "online"[常用字段:智能] OR "数字"[常用字段:智能]	2,172,030
	#4 #1 AND #2 AND #3	68
World Health Organization International Clinical	#1 Title: Postpartum depression OR Postnatal Depression OR Puerperal Depression OR Maternal Depression	159
	#2 Intervention: "cognitive behavior*" OR "cognitive behaviour*" OR "cognitive-behavioral" OR "cognitive-behavioural" OR "cognitive therapy" OR	2,091

Trials Registry Platform (WHO ICTRP)	"behavior* therapy" OR "behaviour* therapy" OR "CBT" OR "CBT-Based" OR "CBT-Counselling" OR "cognitive reorganization" OR "cognitive restructuring" OR "cognitive reconstruction" OR "behavior* treatment" OR "behaviour* treatment" OR "behavior* intervention" OR "behaviour* intervention" OR "behavior* activation" OR "behaviour* activation" OR "behavior* correction" OR "behaviour* correction" OR "behavior* modification" OR "behaviour* modification" OR "behavior* remedy" OR "behaviour* remedy" OR "behavior* adjustment" OR "behaviour* adjustment"	
	#3 #1 AND #2	12
United States National Library of Medicine ClinicalTrials.gov (ClinicalTrials.gov)	#1 Condition/disease: Postpartum depression OR Postnatal Depression OR Puerperal Depression OR Maternal Depression	619
	#2 Intervention/treatment: "cognitive behavior*" OR "cognitive behaviour*" OR "cognitive-behavioral" OR "cognitive-behavioural" OR "cognitive therapy" OR "behavior* therapy" OR "behaviour* therapy" OR "CBT" OR "CBT-Based" OR "CBT-Counselling" OR "cognitive reorganization" OR "cognitive restructuring" OR "cognitive reconstruction" OR "behavior* treatment" OR "behaviour* treatment" OR "behavior* intervention" OR "behaviour* intervention" OR "behavior* activation" OR "behaviour* activation" OR "behavior* correction" OR "behaviour* correction" OR "behavior* modification" OR "behaviour* modification" OR "behavior* remedy" OR "behaviour* remedy" OR "behavior* adjustment" OR "behaviour* adjustment"	8,213
	#3 Other terms: "online" OR "digital" OR "electronic" OR "web" OR "website" OR "internet" OR "computer" OR "mobile application" OR "telephone" OR "internet-based" OR "internet-delivered" OR "web-based" OR "computerized" OR "computer-assisted"	56,743
	#4 #1 AND #2 AND #3	59
ProQuest Dissertations &Theses Database (PQDT)	#1 MAINSUBJECT.EXACT("Postpartum depression")	178
	#2 subject("postnatal depress*") OR subject("postpartum depress*") OR subject("puerperal depress*") OR subject("post-partum depress*") OR subject("post-natal depress*") OR subject("maternal depress*")	842
	#3 #1 OR #2	842
	#4 MAINSUBJECT.EXACT("Behavior modification") OR MAINSUBJECT.EXACT("Cognitive therapy")	13,437
	#5 subject("cognitive behavioral therapy") OR subject("cogniti* behavio* therap*") OR subject("cogniti* behavio* treatment") OR subject("cogniti* behavio* intervention") OR subject("cognitive therap*") OR subject("cognitive treatment") OR subject("cognitive intervention") OR subject("cognition therap*") OR subject("cognition treatment") OR subject("cognition intervention") OR subject("behavio* therap*") OR subject("behavio* treatment") OR subject("behavio* intervention")	14,826
	#6 subject("cogniti* recombination") OR subject("cogniti* regroup") OR subject("cogniti* recombine") OR subject("cogniti* reorganization") OR subject("cogniti* restructuring") OR subject("cogniti* reconstruction") OR subject("behavio* activat*") OR subject("behavio* correct") OR subject("behavio* correction") OR subject("behavio* rectification") OR subject("behavio* reclamation") OR subject("behavio* modification") OR subject("behavio* remedy") OR subject("behavio* adjustment")	1,032
	#7 #4 OR #5 OR #6	15,769
	#8 subject("online") OR subject("digital") OR subject("electronic") OR subject("web") OR subject("website") OR subject("internet") OR subject("computer") OR subject("mobile application") OR subject("telephone")	297,371
	#9 #7 AND #8	693
	#10 subject(ICBT) OR subject(CCBT) OR subject(OCBT)	6
	#11 "internet cognitive behavior therapy" OR "internet cognitive behaviour therapy" OR "internet cognitive behavioral therapy" OR "internet cognitive behavioural therapy" OR "Internet-based cognitive behavior therapy" OR "Internet-based cognitive behaviour therapy" OR "Internet-based cognitive behavioral therapy" OR "Internet-based cognitive behavioural therapy" OR "Internet-delivered cognitive behavior therapy" OR "Internet-delivered cognitive behaviour therapy" OR "Internet-delivered cognitive behavioral therapy" OR "Internet-delivered cognitive behavioural therapy" OR "computerized cognitive behavior therapy" OR "computerized cognitive behaviour therapy" OR "computerized cognitive behavioral therapy" OR "computerized cognitive behavioural therapy" OR "computerized cognitive behavior treatment" OR "computerized cognitive behaviour treatment" OR "computerized cognitive behavioral treatment" OR "computerized cognitive behavioural treatment" OR "online cognitive behavior therapy" OR "online cognitive behaviour therapy" OR "online cognitive behavioral therapy" OR "online cognitive behavioural therapy" OR "Web-based cognitive behavior therapy" OR "Web-based cognitive behaviour therapy" OR "Web-based cognitive behavioral therapy" OR "Web-based cognitive behavioural therapy" OR "Computer-assisted cognitive behavior therapy" OR "Computer-assisted cognitive behaviour therapy" OR "Computer-assisted cognitive behavioral therapy" OR "Computer-assisted cognitive behavioural therapy"	1471
	#12 #9 OR #10 OR #11	2,102
	#13 #3 AND #12	19
Chinese Doctoral Dissertations/Master's Theses Full-text Database (CDFD-CMFD)	#1 (SU = "产后抑郁")	370
	#2 (SU = ("认知" + "行为" + "认知行为" + "CBT" + "认知重组" + "行为激活" + "行为矫正"))	607,382
	#3 (SU = ("网络" + "计算机" + "远程" + "互联网" + "在线" + "平台" + "系统" + "软件" + "程序" + "数字" + "邮件" + "移动" + "线上" + "APP" + "online"))	2,339,208
	#4 #1 AND #2 AND #3	27
China Dissertations	#1 题名或关键词:("产后抑郁")	226

Database (CDDB)	#2 题名或关键词:(“认知”) OR 题名或关键词:(“行为”) OR 题名或关键词:(“认知行为”) OR 题名或关键词:(“CBT”) OR 题名或关键词:(“认知重组”) OR 题名或关键词:(“行为激活”) OR 题名或关键词:(“行为矫正”)	177,231
	#3 题名或关键词(“网络”) OR 题名或关键词:(“计算机”) OR 题名或关键词:(“远程”) OR 题名或关键词:(“互联网”) OR 题名或关键词:(“在线”) OR 题名或关键词:(“平台”) OR 题名或关键词:(“系统”) OR 题名或关键词:(“软件”) OR 题名或关键词:(“程序”) OR 题名或关键词:(“数字”) OR 题名或关键词:(“邮件”) OR 题名或关键词:(“移动”) OR 题名或关键词:(“线上”) OR 题名或关键词:(“APP”) OR 题名或关键词:(“online”)	995,459
	#4 #1 AND #2 AND #3	0

Total results (Search date: from inception to December 31, 2023)

Database/Register	Results
1. PubMed	n=148
2. Embase	n=789
3. CINAHL Plus	n=44
4. Web of Science	n=229
5. The Cochrane Library	n=172
6. PsycINFO	n=37
7. China National Knowledge Infrastructure (CNKI)	n=78
8. WanFang Data Knowledge Service Platform (WanFang Data)	n=22
9. China Science and Technology Journal Database (CQVIP)	n=19
10. Chinese Biomedical Literature Service System (SinoMed)	n=68
11. World Health Organization International Clinical Trials Registry Platform (WHO ICTRP)	n=12
12. United States National Library of Medicine ClinicalTrials.gov (ClinicalTrials.gov)	n=59
13. ProQuest Dissertations &Theses Database (PQDT)	n=19
14. Chinese Doctoral Dissertations/Master’s Theses Full-text Database (CDFD-CMFD)	n=27
15. China Dissertations Database (CDDB)	n=0
Total	N=1723

Supplementary Table S3 Summary of risk of bias assessment

Included Study	Assessors	Risk of Bias																											Overall Bias
		Bias Arising from the Randomization Process				Bias Due to Deviations from Intended Interventions								Bias Due to Missing Outcome Data					Bias in Measurement of the Outcome						Bias in Selection of the Reported Result				
		Q 1.1	Q 1.2	Q 1.3	Domain	Q 2.1	Q 2.2	Q 2.3	Q 2.4	Q 2.5	Q 2.6	Q 2.7	Domain	Q 3.1	Q 3.2	Q 3.3	Q 3.4	Domain	Q 4.1	Q 4.2	Q 4.3	Q 4.4	Q 4.5	Domain	Q 5.1	Q 5.2	Q 5.3	Domain	
O'Mahen 2013	1	NI	PY	N	Low	NI	NI	NI	NA	NA	Y	NA	Some concerns	N	Y	NA	NA	Low	N	N	Y	PY	PN	Some concerns	NI	N	N	Some concerns	Some concerns
	2	Y	Y	N	Low	PY	PY	N	NA	NA	Y	NA	Low	PN	Y	NA	NA	Low	N	N	Y	Y	Y	High	NI	N	N	Some concerns	High
	Final Result	Y	Y	N	Low	PY	PY	PN	NA	NA	Y	NA	Low	N	Y	NA	NA	Low	N	N	Y	PY	PN	Some concerns	NI	N	N	Some concerns	Some concerns
O'Mahen 2014	1	Y	Y	N	Low	PN	PN	NA	NA	NA	Y	NA	Low	N	PY	NA	NA	Low	N	N	PY	PY	PN	Some concerns	NI	PY	N	High	High
	2	Y	Y	NI	Low	PY	PY	N	NA	NA	Y	NA	Low	Y	NA	NA	NA	Low	N	N	Y	Y	Y	High	NI	N	N	Some concerns	High
	Final Result	Y	Y	N	Low	PY	PY	PN	NA	NA	Y	NA	Low	N	PY	NA	NA	Low	N	N	Y	PY	PN	Some concerns	NI	N	N	Some concerns	Some concerns
Ngai 2015	1	Y	Y	N	Low	Y	Y	NI	NA	NA	Y	NA	Some concerns	Y	NA	NA	NA	Low	N	N	Y	PY	PN	Some concerns	Y	N	N	Low	Some concerns
	2	Y	Y	N	Low	Y	Y	N	NA	NA	Y	NA	Low	Y	NA	NA	NA	Low	N	N	Y	Y	Y	High	Y	N	N	Low	High
	Final Result	Y	Y	N	Low	Y	Y	PN	NA	NA	Y	NA	Low	Y	NA	NA	NA	Low	N	N	Y	PY	PN	Some concerns	Y	N	N	Low	Some concerns
Milgrom 2016	1	Y	Y	NI	Low	Y	Y	PN	NA	NA	Y	NA	Low	Y	NA	NA	NA	Low	N	N	Y	PY	PN	Some concerns	Y	N	N	Low	Some concerns
	2	Y	Y	NI	Low	Y	PY	N	NA	NA	Y	NA	Low	Y	NA	NA	NA	Low	N	N	N	NA	NA	Low	Y	N	N	Low	Low
	Final Result	Y	Y	NI	Low	Y	PY	N	NA	NA	Y	NA	Low	Y	NA	NA	NA	Low	N	N	Y	PY	PN	Some concerns	Y	N	N	Low	Some concerns
Pugh 2016	1	Y	Y	N	Low	Y	Y	PN	NA	NA	Y	NA	Low	N	PY	NA	NA	Low	N	N	Y	PY	PN	Some concerns	Y	N	N	Low	Some concerns
	2	Y	Y	N	Low	Y	PY	N	NA	NA	Y	NA	Low	Y	NA	NA	NA	Low	N	N	Y	PY	PY	High	Y	N	N	Low	High
	Final Result	Y	Y	N	Low	Y	PY	N	NA	NA	Y	NA	Low	N	PY	NA	NA	Low	N	N	Y	PY	PN	Some concerns	Y	N	N	Low	Some concerns
Wozney 2017	1	Y	Y	N	Low	PY	PY	PN	NA	NA	PY	NA	Low	N	PY	NA	NA	Low	N	N	Y	PY	PN	Some concerns	NI	N	N	Some concerns	Some concerns
	2	Y	Y	N	Low	PY	PY	N	NA	NA	Y	NA	Low	PY	NA	NA	NA	Low	N	N	NI	Y	Y	High	Y	N	N	Low	High
	Final Result	Y	Y	N	Low	PY	PY	N	NA	NA	PY	NA	Low	N	PY	NA	NA	Low	N	N	PY	PY	PN	Some concerns	NI	N	N	Some concerns	Some concerns

Included Study	Assessors	Risk of Bias																											Overall Bias
		Bias Arising from the Randomization Process				Bias Due to Deviations from Intended Interventions								Bias Due to Missing Outcome Data					Bias in Measurement of the Outcome						Bias in Selection of the Reported Result				
		Q 1.1	Q 1.2	Q 1.3	Domain	Q 2.1	Q 2.2	Q 2.3	Q 2.4	Q 2.5	Q 2.6	Q 2.7	Domain	Q 3.1	Q 3.2	Q 3.3	Q 3.4	Domain	Q 4.1	Q 4.2	Q 4.3	Q 4.4	Q 4.5	Domain	Q 5.1	Q 5.2	Q 5.3	Domain	
Loughnan 2019	1	NI	Y	N	Low	Y	PY	PN	NA	NA	Y	NA	Low	N	Y	NA	NA	Low	N	N	Y	PY	PN	Some concerns	Y	N	N	Low	Some concerns
	2	Y	Y	N	Low	Y	PY	N	NA	NA	Y	NA	Low	Y	NA	NA	NA	Low	N	N	Y	Y	Y	High	Y	N	N	Low	High
	Final Result	NI	Y	N	Low	Y	PY	N	NA	NA	Y	NA	Low	N	Y	NA	NA	Low	N	N	Y	PY	PN	Some concerns	Y	N	N	Low	Some concerns
Fonseca 2020	1	Y	Y	NI	Low	PY	PY	PN	NA	NA	Y	NA	Low	N	Y	NA	NA	Low	N	N	Y	PY	PN	Some concerns	Y	N	N	Low	Some concerns
	2	Y	Y	N	Low	PY	N	N	NA	NA	Y	NA	Low	Y	NA	NA	NA	Low	N	N	Y	Y	Y	High	Y	N	N	Low	High
	Final Result	Y	Y	NI	Low	PY	N	N	NA	NA	Y	NA	Low	N	Y	NA	NA	Low	N	N	Y	PY	PN	Some concerns	Y	N	N	Low	Some concerns
Jannati 2020	1	Y	Y	NI	Low	Y	Y	PN	NA	NA	PN	NI	High	Y	NA	NA	NA	Low	N	N	Y	PY	PN	Some concerns	Y	N	N	Low	High
	2	Y	Y	N	Low	PY	NI	N	NA	NA	PN	NI	High	Y	NA	NA	NA	Low	N	N	Y	PY	PY	High	Y	N	N	Low	High
	Final Result	Y	Y	NI	Low	PY	NI	N	NA	NA	PN	NI	High	Y	NA	NA	NA	Low	N	N	Y	PY	PN	Some concerns	Y	N	N	Low	High
Milgrom 2021	1	Y	Y	N	Low	Y	Y	PN	NA	NA	Y	NA	Low	N	Y	NA	NA	Low	N	N	Y	PY	PN	Some concerns	Y	N	N	Low	Some concerns
	2	Y	Y	N	Low	Y	Y	N	NA	NA	Y	NA	Low	Y	NA	NA	NA	Low	N	N	N	NA	NA	Low	Y	N	N	Low	Low
	Final Result	Y	Y	N	Low	Y	Y	N	NA	NA	Y	NA	Low	N	Y	NA	NA	Low	N	N	Y	PY	PN	Some concerns	Y	N	N	Low	Some concerns
Van 2021	1	Y	Y	PN	Low	PY	PY	PN	NA	NA	Y	NA	Low	N	Y	NA	NA	Low	N	N	Y	PY	PN	Some concerns	Y	N	N	Low	Some concerns
	2	Y	Y	N	Low	PY	PY	N	NA	NA	Y	NA	Low	Y	NA	NA	NA	Low	N	N	Y	Y	Y	High	Y	N	N	Low	High
	Final Result	Y	Y	PN	Low	PY	PY	N	NA	NA	Y	NA	Low	N	Y	NA	NA	Low	N	N	Y	PY	PN	Some concerns	Y	N	N	Low	Some concerns
Seo 2022	1	Y	Y	N	Low	PY	PY	PN	NA	NA	N	NI	High	N	PN	PN	NA	Low	N	N	Y	PY	PN	Some concerns	NI	N	N	Some concerns	Some concerns
	2	Y	Y	N	Low	PY	PY	N	NA	NA	PN	NI	High	Y	NA	NA	NA	Low	N	N	Y	Y	Y	High	Y	N	N	Low	Low
	Final Result	Y	Y	N	Low	PY	PY	N	NA	NA	PN	NI	High	N	PN	PN	NA	Low	N	N	Y	PY	PN	Some concerns	NI	N	N	Some concerns	Some concerns
Shariatpanahi 2022	1	Y	Y	NI	Low	PY	PY	PN	NA	NA	Y	NA	Low	NI	Y	NA	NA	Low	N	N	Y	PY	PN	Some concerns	NI	N	N	Some concerns	Some concerns
	2	Y	Y	N	Low	Y	Y	N	NA	NA	PN	NI	High	Y	NA	NA	NA	Low	N	N	Y	Y	Y	High	Y	N	N	Low	High
	Final Result	Y	Y	NI	Low	PY	PY	N	NA	NA	PY	NA	Low	NI	Y	NA	NA	Low	N	N	Y	PY	PN	Some concerns	NI	N	N	Some concerns	Some concerns

Included Study	Assessors	Risk of Bias																											Overall Bias
		**Bias Arising from the Randomization Process**				**Bias Due to Deviations from Intended Interventions**								**Bias Due to Missing Outcome Data**					**Bias in Measurement of the Outcome**						**Bias in Selection of the Reported Result**				
		**Q 1.1**	**Q 1.2**	**Q 1.3**	**Domain**	**Q 2.1**	**Q 2.2**	**Q 2.3**	**Q 2.4**	**Q 2.5**	**Q 2.6**	**Q 2.7**	**Domain**	**Q 3.1**	**Q 3.2**	**Q 3.3**	**Q 3.4**	**Domain**	**Q 4.1**	**Q 4.2**	**Q 4.3**	**Q 4.4**	**Q 4.5**	**Domain**	**Q 5.1**	**Q 5.2**	**Q 5.3**	**Domain**	
Carona 2023(a)	1	Y	Y	PN	Low	Y	Y	PN	NA	NA	Y	NA	Low	N	Y	NA	NA	Low	N	N	Y	PY	PN	Some concerns	Y	N	N	Low	Some concerns
	2	Y	Y	N	Low	PY	N	N	NA	NA	Y	NA	Low	Y	NA	NA	NA	Low	N	N	Y	Y	Y	High	Y	N	N	Low	High
	Final Result	Y	Y	N	Low	PY	N	N	NA	NA	Y	NA	Low	N	Y	NA	NA	Low	N	N	Y	PY	PN	Some concerns	Y	N	N	Low	Some concerns
Carona 2023(b)	1	Y	Y	PN	Low	Y	Y	PN	NA	NA	Y	NA	Low	N	Y	NA	NA	Low	N	N	Y	PY	PN	Some concerns	Y	N	N	Low	Some concerns
	2	Y	Y	N	Low	PY	N	N	NA	NA	Y	NA	Low	Y	NA	NA	NA	Low	N	N	Y	Y	Y	High	Y	N	N	Low	High
	Final Result	Y	Y	N	Low	PY	N	N	NA	NA	Y	NA	Low	N	Y	NA	NA	Low	N	N	Y	PY	PN	Some concerns	Y	N	N	Low	Some concerns
Huh 2023	1	Y	PY	N	Low	Y	Y	PN	NA	NA	Y	NA	Low	N	Y	NA	NA	Low	N	N	Y	PY	PN	Some concerns	Y	N	N	Low	Some concerns
	2	Y	NI	N	Some concerns	Y	Y	N	NA	NA	Y	NA	Low	Y	NA	NA	NA	Low	N	N	Y	Y	Y	High	Y	N	N	Low	High
	Final Result	Y	PY	N	Low	Y	Y	N	NA	NA	Y	NA	Low	N	Y	NA	NA	Low	N	N	Y	PY	PN	Some concerns	Y	N	N	Low	Some concerns
Merza 2023	1	Y	Y	PN	Low	PY	PY	PN	NA	NA	Y	NA	Low	N	Y	NA	NA	Low	N	N	Y	PY	PN	Some concerns	Y	N	N	Low	Some concerns
	2	Y	Y	N	Low	Y	Y	N	NA	NA	Y	NA	Low	Y	NA	NA	NA	Low	N	N	Y	Y	Y	High	Y	N	N	Low	High
	Final Result	Y	Y	N	Low	Y	Y	N	NA	NA	Y	NA	Low	N	Y	NA	NA	Low	N	N	Y	PY	PN	Some concerns	Y	N	N	Low	Some concerns
Babiy 2024	1	Y	Y	N	Low	Y	PY	PN	NA	NA	Y	NA	Low	N	Y	NA	NA	Low	N	N	Y	PY	PN	Some concerns	Y	N	N	Low	Some concerns
	2	Y	Y	N	Low	Y	Y	N	NA	NA	Y	NA	Low	Y	NA	NA	NA	Low	N	N	Y	Y	Y	High	Y	N	N	Low	High
	Final Result	Y	Y	N	Low	Y	PY	N	NA	NA	Y	NA	Low	N	Y	NA	NA	Low	N	N	Y	PY	PN	Some concerns	Y	N	N	Low	Some concerns

Notes:

①Answers to signalling questions: Yes (Y), Probably Yes (PY), Probably No (PN), No (N), No Information (NI) or Not Applicable (NA).

②Answers to the risk of bias: Low risk of bias (Low), Some concerns or High risk of bias (High).

Supplementary Table S4 Summary of the results of sensitivity analyses

Outcomes	Subgroups	Removed study	Statistical results
1. Depressive symptom	/	Babiy et al., 2024	MD: -1.30 [-2.04, -0.56], P＜0.00001, I^2=99%
		Carona et al., 2023(a)	MD: -1.30 [-2.07, -0.53], P＜0.00001, I^2=99%
		Carona et al., 2023(b)	MD: -0.86 [-1.17, -0.56], P＜0.00001, I^2=93%
		Fonseca et al., 2020	MD: -1.34 [-2.04, -0.63], P＜0.00001, I^2=99%
		Huh et al., 2023	MD: -1.10 [-1.76, -0.43], P＜0.00001, I^2=99%
		Jannati et al., 2020	MD: -1.13 [-1.81, -0.46], P＜0.00001, I^2=99%
		Loughnan et al., 2019	MD: -1.27 [-1.97, -0.56], P＜0.00001, I^2=99%
		Merza et al., 2023	MD: -1.27 [-1.99, -0.56], P＜0.00001, I^2=99%
		Milgrom et al., 2016	MD: -1.27 [-1.96, -0.58], P＜0.00001, I^2=99%
		Milgrom et al., 2021	MD: -1.28 [-1.98, -0.58], P＜0.00001, I^2=99%
		O'Mahen et al., 2013	MD: -1.29 [-2.03, -0.56], P＜0.00001, I^2=99%
		O'Mahen et al., 2014	MD: -1.28 [-1.98, -0.59], P＜0.00001, I^2=99%
		Pugh et al., 2016	MD: -1.26 [-1.95, -0.57], P＜0.00001, I^2=99%
		Seo et al., 2022	MD: -1.30 [-2.00, -0.60], P＜0.00001, I^2=99%
		Shariatpanahi et al., 2022	MD: -1.32 [-2.02, -0.62], P＜0.00001, I^2=99%
		Van et al., 2021	MD: -1.30 [-2.04, -0.55], P＜0.00001, I^2=99%
2. Depressive symptom	Subgroup 1: Western culture	Babiy et al., 2024	MD: -1.34 [-2.20, -0.49], P＜0.00001, I^2=99%
		Carona et al., 2023(a)	MD: -1.34 [-2.23, -0.45], P＜0.00001, I^2=99%
		Carona et al., 2023(b)	MD: -0.81 [-1.12, -0.50], P＜0.00001, I^2=93%
		Fonseca et al., 2020	MD: -1.38 [-2.19, -0.58], P＜0.00001, I^2=99%
		Huh et al., 2023	MD: -1.09 [-1.85, -0.33], P＜0.00001, I^2=99%
		Loughnan et al., 2019	MD: -1.30 [-2.10, -0.49], P＜0.00001, I^2=99%
		Merza et al., 2023	MD: -1.31 [-2.13, -0.49], P＜0.00001, I^2=99%
		Milgrom et al., 2016	MD: -1.30 [-2.10, -0.51], P＜0.00001, I^2=99%
		Milgrom et al., 2021	MD: -1.31 [-2.11, -0.51], P＜0.00001, I^2=99%
		O'Mahen et al., 2013	MD: -1.33 [-2.18, -0.48], P＜0.00001, I^2=99%
		O'Mahen et al., 2014	MD: -1.32 [-2.12, -0.52], P＜0.00001, I^2=99%
		Pugh et al., 2016	MD: -1.29 [-2.08, -0.49], P＜0.00001, I^2=99%
		Van et al., 2021	MD: -1.33 [-2.19, -0.48], P＜0.00001, I^2=99%
	Subgroup 2: Asian culture	Jannati et al., 2020	MD: -0.24 [-0.54, 0.06], P=0.32, I^2=0%
		Seo et al., 2022	MD: -1.52 [-4.31, 1.26], P＜0.00001, I^2=98%
		Shariatpanahi et al., 2022	MD: -1.68 [-4.16, 0.81], P＜0.00001, I^2=97%
3. Depressive symptom	Subgroup 1: ⩽8weeks	Carona et al., 2023(a)	MD: -2.10 [-4.65, 0.46], P＜0.00001, I^2=100%
		Carona et al., 2023(b)	MD: -0.86 [-1.48, -0.23], P＜0.00001, I^2=95%
		Fonseca et al., 2020	MD: -2.20 [-4.51, 0.12], P＜0.00001, I^2=100%
		Jannati et al., 2020	MD: -1.59 [-3.61, 0.43], P＜0.00001, I^2=100%
		Loughnan et al., 2019	MD: -2.00 [-4.20, 0.21], P＜0.00001, I^2=100%

		Seo et al., 2022	MD: -2.09 [-4.21, 0.03], $P<0.00001$, $I^2=100\%$
	Subgroup 2: ≥9weeks	Babiy et al., 2024	MD: -0.95 [-1.40, -0.49], $P<0.00001$, $I^2=93\%$
		Huh et al., 2023	MD: -0.56 [-0.70, -0.42], $P=0.09$, $I^2=41\%$
		Merza et al., 2023	MD: -0.89 [-1.33, -0.46], $P<0.00001$, $I^2=94\%$
		Milgrom et al., 2016	MD: -0.89 [-1.30, -0.48], $P<0.00001$, $I^2=94\%$
		Milgrom et al., 2021	MD: -0.90 [-1.32, -0.48], $P<0.00001$, $I^2=94\%$
		O'Mahen et al., 2013	MD: -0.93 [-1.39, -0.47], $P<0.00001$, $I^2=94\%$
		O'Mahen et al., 2014	MD: -0.91 [-1.33, -0.49], $P<0.00001$, $I^2=94\%$
		Pugh et al., 2016	MD: -0.87 [-1.27, -0.46], $P<0.00001$, $I^2=94\%$
		Shariatpanahi et al., 2022	MD: -0.97 [-1.38, -0.56], $P<0.00001$, $I^2=93\%$
		Van et al., 2021	MD: -0.93 [-1.40, -0.47], $P<0.00001$, $I^2=93\%$
4. Depressive symptom	Subgroup 1: ≤8times	Babiy et al., 2024	MD: -1.51 [-2.68, -0.34], $P<0.00001$, $I^2=99\%$
		Carona et al., 2023(a)	MD: -1.51 [-2.74, -0.28], $P<0.00001$, $I^2=99\%$
		Carona et al., 2023(b)	MD: -0.77 [-1.08, -0.45], $P<0.00001$, $I^2=90\%$
		Fonseca et al., 2020	MD: -1.56 [-2.65, -0.48], $P<0.00001$, $I^2=99\%$
		Jannati et al., 2020	MD: -1.23 [-2.25, -0.20], $P<0.00001$, $I^2=99\%$
		Loughnan et al., 2019	MD: -1.45 [-2.53, -0.38], $P<0.00001$, $I^2=99\%$
		Milgrom et al., 2016	MD: -1.46 [-2.51, -0.41], $P<0.00001$, $I^2=99\%$
		Milgrom et al., 2021	MD: -1.47 [-2.53, -0.41], $P<0.00001$, $I^2=99\%$
		Pugh et al., 2016	MD: -1.43 [-2.48, -0.39], $P<0.00001$, $I^2=99\%$
		Van et al., 2021	MD: -1.50 [-2.67, -0.32], $P<0.00001$, $I^2=99\%$
	Subgroup 2: 9-12times	Huh et al., 2023	MD: -0.64 [-0.80, -0.47], $P=0.38$, $I^2=0\%$
		Merza et al., 2023	MD: -1.54 [-3.17, 0.09], $P<0.00001$, $I^2=98\%$
		O'Mahen et al., 2013	MD: -1.63 [-3.21, -0.06], $P<0.00001$, $I^2=98\%$
		O'Mahen et al., 2014	MD: -1.58 [-2.89, -0.28], $P<0.00001$, $I^2=98\%$
	Subgroup 3: 13-16times	Shariatpanahi et al., 2022	/
5. Depressive symptom	Subgroup 1: ≤1hour	Carona et al., 2023(a)	MD: -3.26 [-7.32, 0.79], $P<0.00001$, $I^2=100\%$
		Carona et al., 2023(b)	MD: -1.19 [-1.96, -0.42], $P<0.00001$, $I^2=96\%$
		Jannati et al., 2020	MD: -2.41 [-5.17, 0.35], $P<0.00001$, $I^2=100\%$
		O'Mahen et al., 2013	MD: -3.22 [-7.41, 0.97], $P<0.00001$, $I^2=100\%$
	Subgroup 2: >1hour	Babiy et al., 2024	MD: -1.57 [-2.88, -0.27], $P<0.00001$, $I^2=98\%$
		Huh et al., 2023	MD: -0.55 [-0.75, -0.34], $P=0.09$, $I^2=59\%$
		Merza et al., 2023	MD: -1.42 [-2.54, -0.30], $P<0.00001$, $I^2=98\%$
		Van et al., 2021	MD: -1.54 [-2.88, -0.19], $P<0.00001$, $I^2=98\%$
6. Depressive symptom	Subgroup 1: Website	Carona et al., 2023(a)	MD: -1.37 [-2.86, 0.13], $P<0.00001$, $I^2=99\%$
		Carona et al., 2023(b)	MD: -0.57 [-0.80, -0.33], $P=0.0003$, $I^2=75\%$
		Fonseca et al., 2020	MD: -1.43 [-2.74, -0.12], $P<0.00001$, $I^2=99\%$
		Loughnan et al., 2019	MD: -1.31 [-2.60, -0.02], $P<0.00001$, $I^2=99\%$

		Milgrom et al., 2016	MD: -1.31 [-2.56, -0.07], P＜0.00001, I^2=99%
		Milgrom et al., 2021	MD: -1.33 [-2.59, -0.06], P＜0.00001, I^2=99%
		O'Mahen et al., 2013	MD: -1.35 [-2.78, 0.07], P＜0.00001, I^2=99%
		O'Mahen et al., 2014	MD: -1.34 [-2.60, -0.07], P＜0.00001, I^2=99%
		Pugh et al., 2016	MD: -1.29 [-2.53, -0.04], P＜0.00001, I^2=99%
	Subgroup 2: APP&Telegram	Jannati et al., 2020	MD: -0.24 [-0.54, 0.06], P=0.32, I^2=0%
		Seo et al., 2022	MD: -1.52 [-4.31, 1.26], P＜0.00001, I^2=98%
		Shariatpanahi et al., 2022	MD: -1.68 [-4.16, 0.81], P＜0.00001, I^2=97%
	Subgroup 3: Zoom	Babiy et al., 2024	MD: -1.57 [-2.88, -0.27], P＜0.00001, I^2=98%
		Huh et al., 2023	MD: -0.55 [-0.75, -0.34], P=0.09, I^2=59%
		Merza et al., 2023	MD: -1.42 [-2.54, -0.30], P＜0.00001, I^2=98%
		Van et al., 2021	MD: -1.54 [-2.88, -0.19], P＜0.00001, I^2=98%
7. Depressive symptom	Subgroup 1: ≤8times	Babiy et al., 2024	MD: -1.51 [-2.68, -0.34], P＜0.00001, I^2=99%
		Carona et al., 2023(a)	MD: -1.51 [-2.74, -0.28], P＜0.00001, I^2=99%
		Carona et al., 2023(b)	MD: -0.77 [-1.08, -0.45], P＜0.00001, I^2=90%
		Fonseca et al., 2020	MD: -1.56 [-2.65, -0.48], P＜0.00001, I^2=99%
		Jannati et al., 2020	MD: -1.23 [-2.25, -0.20], P＜0.00001, I^2=99%
		Loughnan et al., 2019	MD: -1.45 [-2.53, -0.38], P＜0.00001, I^2=99%
		Milgrom et al., 2016	MD: -1.46 [-2.51, -0.41], P＜0.00001, I^2=99%
		Milgrom et al., 2021	MD: -1.47 [-2.53, -0.41], P＜0.00001, I^2=99%
		Pugh et al., 2016	MD: -1.43 [-2.48, -0.39], P＜0.00001, I^2=99%
		Van et al., 2021	MD: -1.50 [-2.67, -0.32], P＜0.00001, I^2=99%
	Subgroup 2: 9-12times	Huh et al., 2023	MD: -0.64 [-0.80, -0.47], P=0.38, I^2=0%
		Merza et al., 2023	MD: -1.54 [-3.17, 0.09], P＜0.00001, I^2=98%
		O'Mahen et al., 2013	MD: -1.63 [-3.21, -0.06], P＜0.00001, I^2=98%
		O'Mahen et al., 2014	MD: -1.58 [-2.89, -0.28], P＜0.00001, I^2=98%
8. Depressive symptom	Subgroup 1: Personal	Carona et al., 2023(a)	MD: -1.31 [-2.47, -0.15], P＜0.00001, I^2=99%
		Carona et al., 2023(b)	MD: -0.71 [-1.02, -0.40], P＜0.00001, I^2=88%
		Fonseca et al., 2020	MD: -1.36 [-2.39, -0.32], P＜0.00001, I^2=99%
		Jannati et al., 2020	MD: -1.08 [-2.06, -0.11], P＜0.00001, I^2=99%
		Loughnan et al., 2019	MD: -1.26 [-2.29, -0.24], P＜0.00001, I^2=99%
		Milgrom et al., 2016	MD: -1.27 [-2.27, -0.27], P＜0.00001, I^2=99%
		Milgrom et al., 2021	MD: -1.28 [-2.29, -0.27], P＜0.00001, I^2=99%
		O'Mahen et al., 2013	MD: -1.30 [-2.40, -0.19], P＜0.00001, I^2=99%
		O'Mahen et al., 2014	MD: -1.29 [-2.29, -0.28], P＜0.00001, I^2=99%
		Pugh et al., 2016	MD: -1.25 [-2.24, -0.26], P＜0.00001, I^2=99%
		Seo et al., 2022	MD: -1.31 [-2.32, -0.30], P＜0.00001, I^2=99%
		Shariatpanahi et al., 2022	MD: -1.34 [-2.35, -0.32], P＜0.00001, I^2=99%
	Subgroup 2: Team	Babiy et al., 2024	MD: -1.57 [-2.88, -0.27], P＜0.00001, I^2=98%
		Huh et al., 2023	MD: -0.55 [-0.75, -0.34], P=0.09, I^2=59%
		Merza et al., 2023	MD: -1.42 [-2.54, -0.30], P＜0.00001, I^2=98%
		Van et al., 2021	MD: -1.54 [-2.88, -0.19], P＜0.00001, I^2=98%

9. Depressive symptom	Subgroup 1: Yes	Babiy et al., 2024	MD: -0.95 [-1.40, -0.49], $P<0.00001$, I^2=93%
		Huh et al., 2023	MD: -0.56 [-0.70, -0.42], $P<=0.09$, I^2=41%
		Merza et al., 2023	MD: -0.89 [-1.33, -0.46], $P<0.00001$, I^2=94%
		Milgrom et al., 2016	MD: -0.89 [-1.30, -0.48], $P<0.00001$, I^2=94%
		Milgrom et al., 2021	MD: -0.90 [-1.32, -0.48], $P<0.00001$, I^2=94%
		O'Mahen et al., 2013	MD: -0.93 [-1.39, -0.47], $P<0.00001$, I^2=94%
		O'Mahen et al., 2014	MD: -0.91 [-1.33, -0.49], $P<0.00001$, I^2=94%
		Pugh et al., 2016	MD: -0.87 [-1.27, -0.46], $P<0.00001$, I^2=94%
		Shariatpanahi et al., 2022	MD: -0.97 [-1.38, -0.56], $P<0.00001$, I^2=93%
		Van et al., 2021	MD: -0.93 [-1.40, -0.47], $P<0.00001$, I^2=93%
	Subgroup 2: No	Carona et al., 2023(a)	MD: -2.10 [-4.65, 0.46], $P<0.00001$, I^2=100%
		Carona et al., 2023(b)	MD: -0.86 [-1.48, -0.23], $P<0.00001$, I^2=95%
		Fonseca et al., 2020	MD: -2.20 [-4.51, 0.12], $P<0.00001$, I^2=100%
		Jannati et al., 2020	MD: -1.59 [-3.61, 0.43], $P<0.00001$, I^2=100%
		Loughnan et al., 2019	MD: -2.00 [-4.20, 0.21], $P<0.00001$, I^2=100%
		Seo et al., 2022	MD: -2.09 [-4.21, 0.03], $P<0.00001$, I^2=100%

Supplementary Table S5 Differences between protocol and review

Changed part	Protocal	Review	Description and Reason for change
Title	"efficiency"	"Effects"	We thought the expression "effects" is more accurate and authentic.
Search strategy	Conduct a preliminary search in China National Knowledge Infrastructure (CNKI) to find all Chinese subject words and free words that can identify potential target documents.	Conduct a preliminary search in Chinese Biomedical Literature Service System (SinoMed) to find all Chinese subject words and free words that can identify potential target documents.	In the SinoMed, subject words are more systematic and comprehensive.
Search strategy	Conduct a formal search in PsycINFO, PsycArticles.	Conduct a formal search in PsycINFO.	The PsycINFO database is a prestigious resource provided by the American Psychological Association (APA) that contains academic journal articles, book catalogs, book abstracts, and monographs, and is the world’s largest resource for peer-reviewed literature in the behavioral sciences and mental health.
Search strategy	Conduct a formal search in Cochrane Controlled Clinical Trials Center Registry (CENTRAL).	Conduct a formal search in The Cochrane library.	The Cochrane Library is the main product of the International Cochrane Collaboration, and CENTRAL is its sub-database.
Search strategy	Use Google Scholar for supplementary Search.	/	With the help of the librarian, our search strategy has been relatively comprehensive, so we think that manually search and trace the list of all references included in the study is sufficient.
Search strategy	Search date: from inception to January 1, 2024	Search date: from inception to December 31, 2023	Due to the different built-in search principles of each database, the use of "December 31, 2023" expression can make the search results more unified.
Eligibility criteria	Traditional face-to-face cognitive behavioral therapy or other conventional treatment (such as medication, physical therapy or other psychotherapy).	Conventional therapy (e.g., medication, physical therapy, or other psychotherapy).	The purpose of our study was to compare the effects of OCBT versus conventional therapy, not OCBT versus face-to-face CBT.
Eligibility criteria	Studies that did not provide measurements of depression scores before and after the intervention, or provided data that did not translate to measurements.	Studies that did not provide measurements of depression scores before and after the intervention were excluded.	In our review, in order to make the evidence more comprehensive, substantial and reliable, studies that provided the exact measurement of depression scores before and after the intervention in the results were included. And studies whose measurement data could not be translated were obtained by contacting the author via email.
Eligibility criteria	Randomized controlled trials (RCT) and quasi-randomized controlled trials (quasi-RCT).	Randomized controlled trials (RCT)	The RCT has a higher level of evidence.
Data analyses	We will use Revman5.3 software for data analysis.	Data analysis was performed using Revman5.4 software.	We used the latest version of the software to facilitate research.

Study or Subgroup	OCBT Mean	OCBT SD	OCBT Total	Control Mean	Control SD	Control Total	Weight	Std. Mean Difference IV, Random, 95% CI
**2.6.1 ≤1hour**								
Carona 2023(a)	8.75	4.53	267	10.78	5.06	445	12.6%	−0.42 [−0.57, −0.26]
Carona 2023(b)	8.29	0.33	267	10.07	0.25	445	12.5%	−6.29 [−6.65, −5.93]
Jannati 2020	8.18	1.5	38	15.05	2.9	37	12.1%	−2.96 [−3.62, −2.29]
O'Mahen 2013	10.94	5.57	181	14.28	6.63	162	12.6%	−0.55 [−0.76, −0.33]
**Subtotal (95% CI)**			**753**			**1089**	**49.9%**	**−2.55 [−4.89, −0.21]**
Heterogeneity: $Tau^2 = 5.67$; $Chi^2 = 925.08$, df = 3 (P < 0.00001); $I^2 = 100\%$								
Test for overall effect: Z = 2.13 (P = 0.03)								
**2.6.2 > 1hour**								
Babiy 2024	11.37	5.24	202	13.5	5.2	203	12.6%	−0.41 [−0.60, −0.21]
Huh 2023	10.8	0.58	71	12.86	0.6	65	12.3%	−3.47 [−4.01, −2.94]
Merza 2023	10.2	4.32	92	13.96	4.9	91	12.6%	−0.81 [−1.11, −0.51]
Van 2021	11.65	4.83	202	14.04	4.54	201	12.6%	−0.51 [−0.71, −0.31]
**Subtotal (95% CI)**			**567**			**560**	**50.1%**	**−1.25 [−2.07, −0.44]**
Heterogeneity: $Tau^2 = 0.67$; $Chi^2 = 114.81$, df = 3 (P < 0.00001); $I^2 = 97\%$								
Test for overall effect: Z = 3.01 (P = 0.003)								
**Total (95% CI)**			**1320**			**1649**	**100.0%**	**−1.91 [−3.01, −0.82]**
Heterogeneity: $Tau^2 = 2.46$; $Chi^2 = 1068.86$, df = 7 (P < 0.00001); $I^2 = 99\%$								
Test for overall effect: Z = 3.43 (P = 0.0006)								
Test for subgroup differences: $Chi^2 = 1.04$, df = 1 (P = 0.31), $I^2 = 4.3\%$								

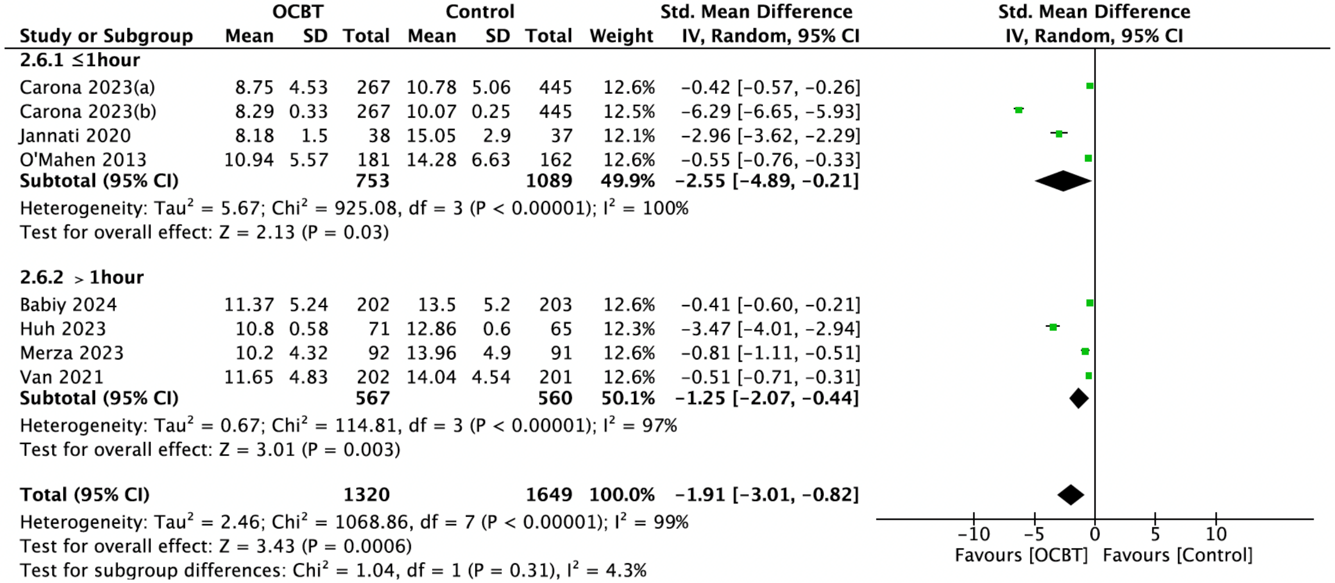

Figure S1 Forest plot of the effect of OCBT on postpartum depression symptom in different each intervention duration [17,24–26,28,29,31,36].

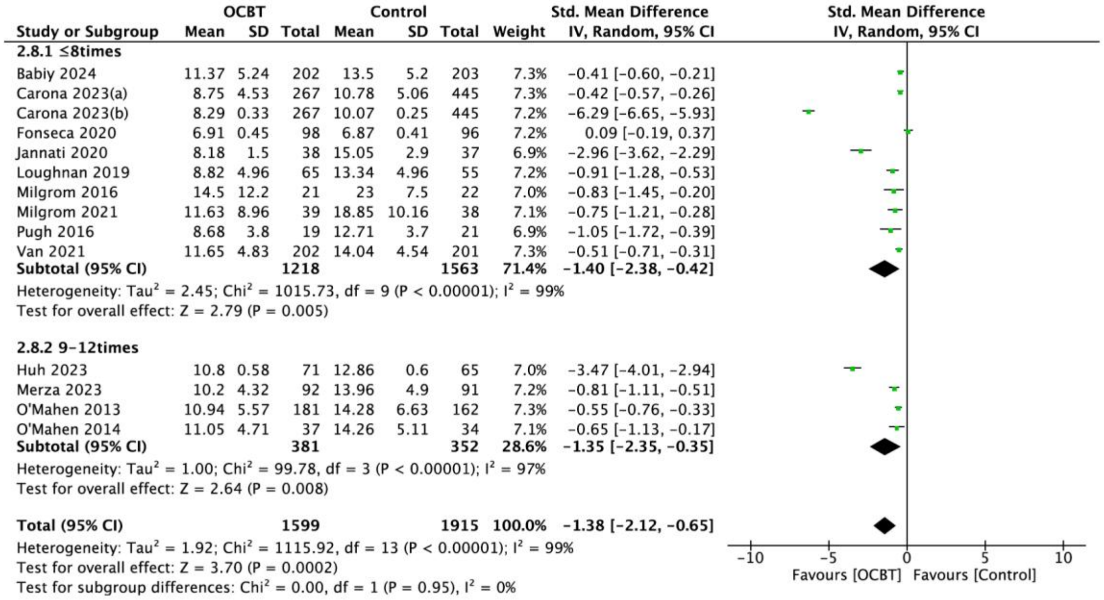

Figure S2 Forest plot of the effect of OCBT on postpartum depression symptom in different intervention module number [17,21,24–29,31,34–38].

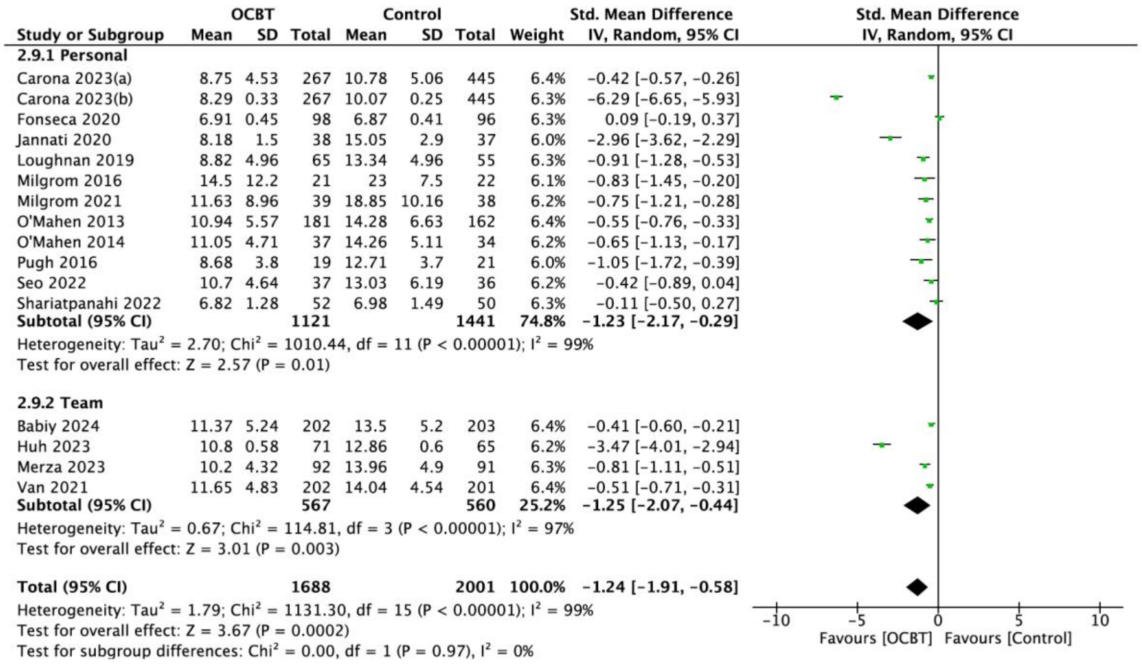

Figure S3. Forest plot of the effect of OCBT on postpartum depression symptom in different intervention form [17,21,22,24–31,34–38].

Supplementary Figure S4 Summary of funnel plots

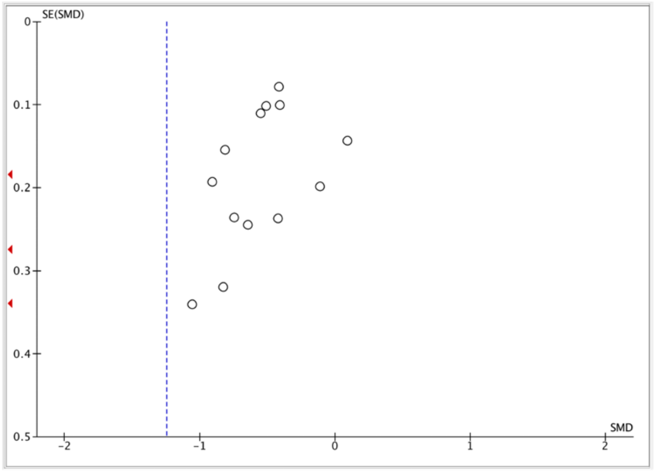

(a) Postpartum depression symptom

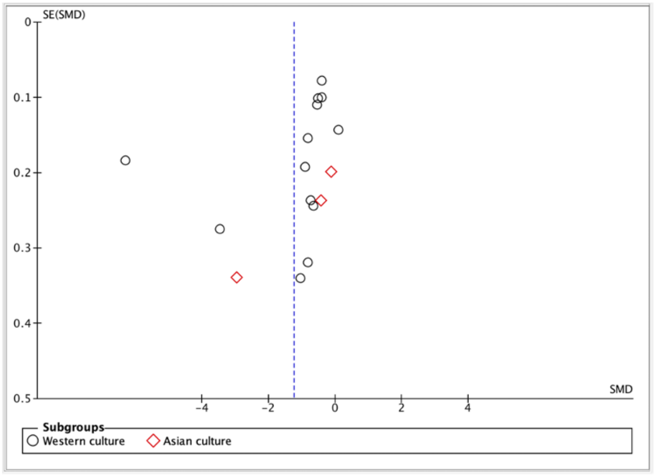

(b) Subgroup 1 -culture

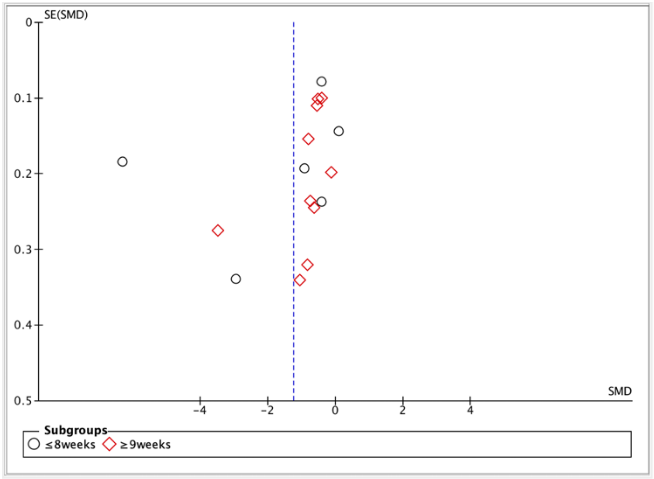

(c) Subgroup 2 -intervention duration

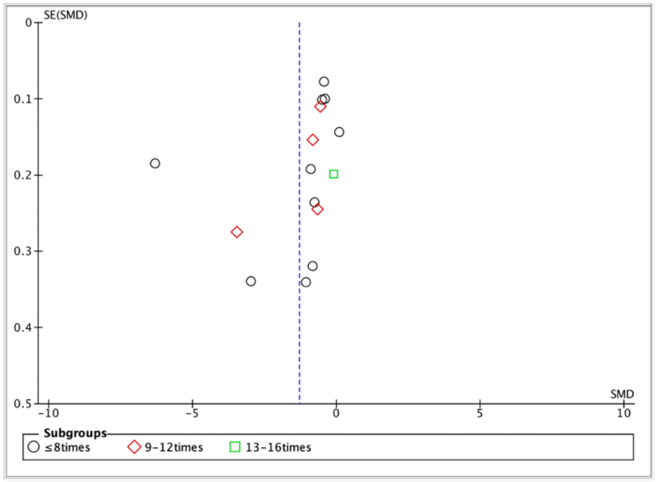

(d) Subgroup 3-intervention number

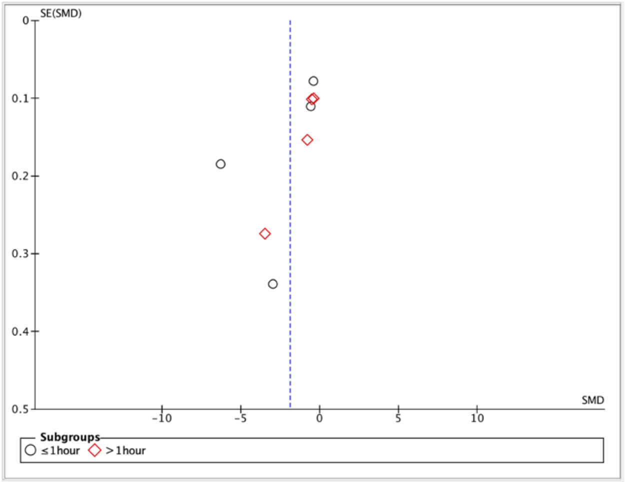

(e) Subgroup 4 -each intervention duration

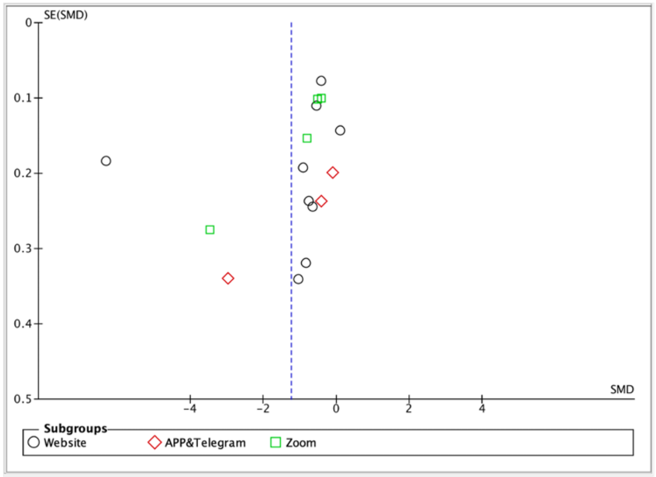

(f) Subgroup 5 -intervention platform

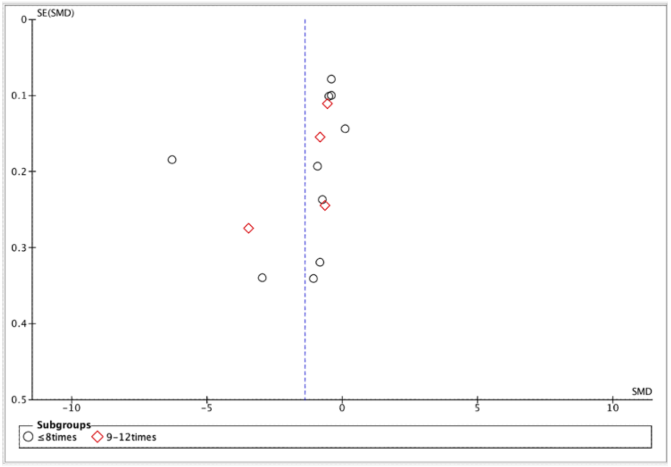

(g) Subgroup 6 -intervention module number

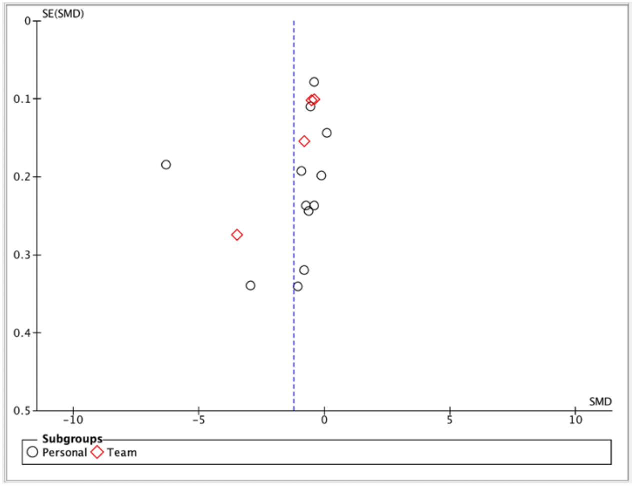

(h) Subgroup 7 -intervention form

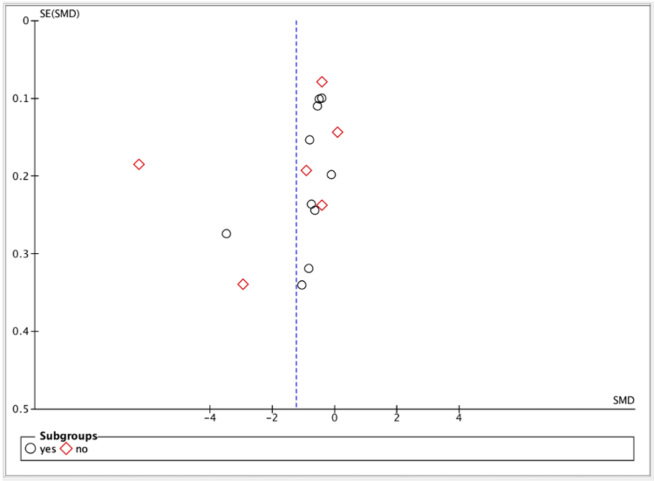

(i) Subgroup 8 -professional guidance

Supplementary Figure S5 GRADE evidence profile

Quality assessment							No of patients		Effect		Quality	Importance
No of studies	Design	Risk of bias	Inconsistency	Indirectness	Imprecision	Other considerations	OCBT	TAU/WLC	Relative (95% CI)	Absolute		
**Depressive symptom (Better indicated by lower values)**												
16	randomised trials	no serious risk of bias	serious[1]	no serious indirectness	no serious imprecision	reporting bias[2]	1688	2001	-	SMD 1.24 lower (1.91 to 0.58 lower)	⊕⊕OO LOW	IMPORTANT

Quality assessment							No of patients		Effect		Quality	Importance
No of studies	Design	Risk of bias	Inconsistency	Indirectness	Imprecision	Other considerations	OCBT	TAU/WLC (subgroup)	Relative (95% CI)	Absolute		
**Culture - Western culture (Better indicated by lower values)**												
13	randomised trials	no serious risk of bias	serious[1]	no serious indirectness	no serious imprecision	reporting bias[2]	1561	1878	-	SMD 1.27 lower (2.02 to 0.51 lower)	⊕⊕OO LOW	IMPORTANT
**Culture - Asian culture (Better indicated by lower values)**												
3	randomised trials	serious[3]	serious[4]	no serious indirectness[5]	very serious[5]	reporting bias[2]	127	123	-	SMD 1.14 lower (2.62 lower to 0.34 higher)	⊕OOO VERY LOW	IMPORTANT
**Intervention duration - ≤8weeks (Better indicated by lower values)**												
6	randomised trials	no serious risk of bias	serious[1]	no serious indirectness	serious[6]	reporting bias[2]	772	1114	-	SMD 1.81 lower (3.63 lower to 0.01 higher)	⊕OOO VERY LOW	IMPORTANT
**Intervention duration - ≥9weeks (Better indicated by lower values)**												
10	randomised trials	no serious risk of bias	serious[7]	no serious indirectness	no serious imprecision	reporting bias[2]	916	887	-	SMD 0.88 lower (1.27 to 0.5 lower)	⊕⊕OO LOW	IMPORTANT
**Intervention number - ≤8times (Better indicated by lower values)**												
10	randomised trials	no serious risk of bias	serious[1]	no serious indirectness	no serious imprecision	reporting bias[2]	1218	1563	-	SMD 1.4 lower (2.38 to 0.42 lower)	⊕⊕OO LOW	IMPORTANT
**Intervention number - 9-12times (Better indicated by lower values)**												
4	randomised trials	no serious risk of bias	serious[8]	no serious indirectness	no serious imprecision	reporting bias[2]	381	352	-	SMD 1.35 lower (2.35 to 0.35 lower)	⊕⊕OO LOW	IMPORTANT
**Intervention number - 13-16times (Better indicated by lower values)**												
1	randomised trials	no serious risk of bias	no serious inconsistency	no serious indirectness	very serious[9]	none	52	50	-	SMD 0.11 lower (0.5 lower to 0.27 higher)	⊕⊕OO LOW	IMPORTANT
**Each intervention duration - ≤1hour (Better indicated by lower values)**												
4	randomised trials	no serious risk of bias	serious[10]	no serious indirectness	no serious imprecision	reporting bias[2]	753	1089	-	SMD 2.55 lower (4.89 to 0.21 lower)	⊕⊕OO LOW	IMPORTANT
**Each intervention duration - >1hour (Better indicated by lower values)**												
4	randomised trials	no serious risk of bias	serious[8]	no serious indirectness	no serious imprecision	reporting bias[2]	567	560	-	SMD 1.25 lower (2.07 to 0.44 lower)	⊕⊕OO LOW	IMPORTANT

Quality assessment							No of patients		Effect		Quality	Importance
No of studies	Design	Risk of bias	Inconsistency	Indirectness	Imprecision	Other considerations	OCBT	TAU/WLC (subgroup)	Relative (95% CI)	Absolute		
Intervention platform - Website (Better indicated by lower values)												
9	randomised trials	no serious risk of bias	serious[1]	no serious indirectness	no serious imprecision	reporting bias[2]	994	1318	-	SMD 1.26 lower (2.41 to 0.11 lower)	⊕⊕OO LOW	IMPORTANT
Intervention platform - APP&Telegram (Better indicated by lower values)												
3	randomised trials	serious[3]	serious[4]	no serious indirectness	very serious[5]	reporting bias[2]	127	123	-	SMD 1.14 lower (2.62 lower to 0.34 higher)	⊕OOO VERY LOW	IMPORTANT
Intervention platform - Zoom (Better indicated by lower values)												
4	randomised trials	no serious risk of bias	serious[6]	no serious indirectness	no serious imprecision	reporting bias[2]	567	560	-	SMD 1.25 lower (2.07 to 0.44 lower)	⊕⊕OO LOW	IMPORTANT
Intervention module number - ≤8times (Better indicated by lower values)												
10	randomised trials	no serious risk of bias	serious[1]	no serious indirectness	no serious imprecision	reporting bias[2]	1218	1563	-	SMD 1.4 lower (2.38 to 0.42 lower)	⊕⊕OO LOW	IMPORTANT
Intervention module number - 9-12times (Better indicated by lower values)												
4	randomised trials	no serious risk of bias	serious[6]	no serious indirectness	no serious imprecision	reporting bias[2]	381	352	-	SMD 1.35 lower (2.35 to 0.35 lower)	⊕⊕OO LOW	IMPORTANT
Intervention form - Personal (Better indicated by lower values)												
12	randomised trials	no serious risk of bias	serious[1]	no serious indirectness	no serious imprecision	reporting bias[2]	1121	1441	-	SMD 1.23 lower (2.17 to 0.29 lower)	⊕⊕OO LOW	IMPORTANT
Intervention form - Team (Better indicated by lower values)												
4	randomised trials	no serious risk of bias	serious[6]	no serious indirectness	no serious imprecision	reporting bias[2]	567	560	-	SMD 1.25 lower (2.07 to 0.44 lower)	⊕⊕OO LOW	IMPORTANT
Professional guidance - yes (Better indicated by lower values)												
10	randomised trials	no serious risk of bias	serious[7]	no serious indirectness	no serious imprecision	reporting bias[2]	916	887	-	SMD 0.88 lower (1.27 to 0.5 lower)	⊕⊕OO LOW	IMPORTANT
Professional guidance - no (Better indicated by lower values)												
6	randomised trials	no serious risk of bias	serious[1]	no serious indirectness	serious[8]	reporting bias[2]	772	1114	-	SMD 1.81 lower (3.63 lower to 0.01 higher)	⊕OOO VERY LOW	IMPORTANT

Supplementary File S1 Studies ineligible following full-text review (N=57)

Reason 1 for exclusion: ineligible population (n=24)

[1] YANG Juan, WANG Ai. Effects of cognitive behavioral intervention based on WeChat platform combined with interpersonal psychotherapy on the occurrence of postpartum depression and neurotransmitter and mood state of parturients. [J]. Clinical Research and Practice, 2023, 8(15): 189-91.

[2] FU Qinghua, ZHAO Zhongrun, JIA Changqing. The role of online education based on cognitive behavioral therapy in the negative emotions and parent-child behaviors of postpartum depressed mothers. [J]. Maternal and Child Health Care of China. July 2023, Vol. 38, No. 13: 2478-82.

[3] LI Yu, LIU Yi, WANG Wenjuan, et al. Study on the effect of online group psychological intervention on improving maternal bad mood. [J].Journal of Xiangnan University（Medical Sciences）Jun.2023 Vol.25 No.2:24-6.

[4] ACTRN. Online cognitive behaviour therapy for the prevention of postnatal depression in at-risk mothers: a randomised controlled trial [J]. https://trialsearchwhoint/Trial2aspx?TrialID=ACTRN12609001032246, 2009,

[5] D'ANGELO E J, LLERENA-QUINN R, SHAPIRO R, et al. Adaptation of the preventive intervention program for depression for use with predominantly low-income latino families [J]. Family Process, 2009, 48(2): 269-91.

[6] SHEEBER L B, SEELEY J R, FEIL E G, et al. Development and pilot evaluation of an Internet-facilitated cognitive-behavioral intervention for maternal depression [J]. Journal of Consulting and Clinical Psychology, 2012, 80(5): 739-49.

[7] KIM D R, HANTSOO L, THASE M E, et al. Computer-Assisted Cognitive Behavioral Therapy for Pregnant Women with Major Depressive Disorder [J]. J Womens Health, 2014, 23(10): 842-8.

[8] NCT02469324. 2015. A Comparative RCT of Brief Internet-based Compassionate Mind Training and Cognitive-behavioral Therapy for Mothers and Their Babies. https://clinicaltrials.gov/study/NCT02469324

[9] FELDER J N, SEGAL Z, BECK A, et al. An Open Trial of Web-Based Mindfulness-Based Cognitive Therapy for Perinatal Women at Risk for Depressive Relapse [J]. Cognitive and Behavioral Practice, 2017, 24(1): 26-37.

[10] NCT03995316. 2019. Responsive e-Health Intervention for Perinatal Depression in Healthcare Settings (MMB). https://clinicaltrials.gov/study/NCT03995316

[11] SIMHI M, SARID O, ROWE H, et al. A cognitive—behavioral intervention for postpartum anxiety and depression: Individual phone vs. group format [J]. Journal of Clinical Medicine, 2021, 10(24):

[12] SOLNESS C L, KROSKA E B, HOLDEFER P J, et al. Treating postpartum depression in rural veterans using internet delivered CBT: Program evaluation of MomMoodBooster [J]. Journal of Behavioral Medicine, 2021, 44(4): 454-66.

[13] GEMMILL A W, OLIVA J L, ERICKSEN J, et al. Web-based treatment for depression in pregnancy: a feasibility study of Mum2BMoodBooster [J]. BMC Psychiatry, 2022, 22(1):

[14] IRCT20110228005931N. 2022. Effect of Internet cognitive-behavioral psychotherapy with psychological well-being on depression of pregnant women. https://trialsearchwhoint/Trial2aspx?TrialID=IRCT20110228005931N12

[15] QIN X L, LIU C F, ZHU W, et al. Preventing Postpartum Depression in the Early Postpartum Period Using an App-Based Cognitive Behavioral Therapy Program: A Pilot Randomized Controlled Study [J]. International Journal of Environmental Research and Public Health, 2022, 19(24): 13.

[16] SUCHAN V, PEYNENBURG V, THIESSEN D, et al. Transdiagnostic Internet-Delivered Cognitive Behavioral Therapy for Symptoms of Postpartum Anxiety and Depression: Feasibility Randomized Controlled Trial [J]. JMIR Form Res, 2022, 6(9): e37216.

[17] Using an Online e-Health Program to Improve Postpartum Depression [J]. Integrative Medicine Alert, 2023, 26(7): 1-8.

[18] DANAHER B G, SEELEY J R, SILVER R K, et al. Trial of a Patient-Directed eHealth Program to Ameliorate Perinatal Depression: The MomMoodBooster2 Practical Effectiveness Study [J]. Obstetrical and Gynecological Survey, 2023, 78(10): 557-9.

[19] IRCT20110228005931N. 2023. Comparing the effectiveness of web-based unified transdiagnostic treatment with specific diagnosis. https://trialsearchwhoint/Trial2aspx?TrialID=IRCT20110228005931N13

[20] JIDONG D E, IKE J T, HUSAIN N, et al. Culturally adapted psychological intervention for treating maternal depression in British mothers of African and Caribbean origin: a randomized controlled feasibility trial [J]. Clinical psychology & psychotherapy, 2023, 30(3): 548-65.

[21] OKATSU A, KANIE A, KATAOKA Y. Evaluation of the effect of a midwife-led online program using cognitive behavioral therapy for pregnant women at risk for anxiety disorder in Japan: A pilot randomized controlled trial [J]. PLoS One, 2023, 18(5): e0281632.

[22] POLANCZYK G V, FATORI D, ZUCCOLO P, et al. Smartphone-assisted online brief cognitive behavioral therapy to treat maternal depression: findings of a randomized controlled trial [J]. Brazilian Journal of Psychiatry, 2023, 45(1): 50-3.

[23] MONTEIRO F, PEREIRA M, CANAVARRO M C, et al. Be a mom’s efficacy in enhancing positive mental health among postpartum women presenting low risk for postpartum depression: Results from a pilot randomized trial [J]. International Journal of Environmental Research and Public Health, 2020, 17(13): 1-19.

[24] SUCHAN V A M. Examining the acceptability and effectiveness of transdiagnostic, internet-delivered cognitive behaviour therapy for symptoms of postpartum anxiety and depression: A randomized controlled trial [D]; ProQuest Information & Learning, 2022.

Reason 2 for exclusion: Clinical registration trial results has been published and the literature was included (n=9)

[1] NCT00795652. 2008. Mom: managing Our Mood, Part of The Family Help Program. https://clinicaltrials.gov/study/NCT00795652

[2] ACTRN12613000881730. 2013. Web-based Cognitive Behavioural Therapy for Women With Postnatal Depression: a Comparison with Face-to-Face Therapy. https://trialsearchwhoint/Trial2aspx?TrialID=ACTRN12613000881730

[3] ACTRN12613000113752. 2013. Web-based Cognitive Behavioural Therapy for Women with Postnatal Depression. https://trialsearchwhoint/Trial2aspx?TrialID=ACTRN12613000113752

[4] ACTRN12616000559415. 2016. A randomised controlled trial comparing the clinical efficacy of internet-delivered cognitive behavioural therapy for perinatal anxiety and depression to treatment as usual. Study 2: the Perinatal MUMentum Program: postpartum Course. https://trialsearchwhoint/Trial2aspx?TrialID=ACTRN12616000559415

[5] NCT 01507649. 2012. Effect of a Telephone-based Intervention on Postnatal Depression. https://clinicaltrials.gov/study/NCT01507649
[6] NCT 00942721. 2009. Cognitive Behavioral Therapy Delivered Over the Internet for Women With Postpartum Depression. https://clinicaltrials.gov/study/NCT00942721
[7] NCT 04913584. 2021. Peer Administered Online CBT for PPD (PL-OCBT). https://clinicaltrials.gov/study/NCT04913584
[8] NCT 04928742. 2021. Online PHN CBT for PPD. https://clinicaltrials.gov/study/NCT04928742
[9] IRCT2017071826484N3. 2017. Comparison of the Effectiveness of Cognitive Behavioral Therapy Based on Virtual Content with Drug Therapy in Postpartum Depression in Improving Weight Indices in Children Up to 4 Months. https://trialsearch.who.int/Trial2.aspx?TrialID=IRCT2017071826484N3

Reason 3 for exclusion: Not RCTs (n=5)

[1] DANAHER B G, MILGROM J, SEELEY J R, et al. MomMoodBooster Web-Based Intervention for Postpartum Depression: Feasibility Trial Results [J]. Journal of Medical Internet Research, 2013, 15(11): 20.
[2] MERZA D, LAYTON H, SAVOY C, et al. Online Peer-Delivered 1-Day Cognitive Behavioral Therapy–Based Workshops for Postpartum Depression: A Pilot Study [J]. Journal of Clinical Psychiatry, 2023, 84(1):
[3] MILGROM J, GEMMILL A. Feasibility and efficacy of an internet treatment for postnatal depression utilising a behavioural activation approach [J]. Evid Based Nurs, 2014, 17(4): 102.
[4] FONSECA A, BRANQUINHO M, CANAVARRO M C. Application of the cognitive-behavior therapy principles in the development of e-mental health tools: The case of Be a Mom, a web-based psychological intervention to prevent postpartum depression [J]. Psychologica, 2020, 63(2): 119-37.
[5] NCT 05077644. 2021. Mobile Application in the Management of Mild to Moderate Postpartum Depression (PPD). https://clinicaltrials.gov/study/NCT05077644

Reason 4 for exclusion: Ineligible intervention (n = 4)

[1] UGARRIZA D N, SCHMIDT L. Telecare for women with postpartum depression [J]. J Psychosoc Nurs Ment Health Serv, 2006, 44(1): 37-45.
[2] BEVAN D, WITTKOWSKI A, WELLS A. A Multiple-Baseline Study of the Effects Associated With Metacognitive Therapy in Postpartum Depression [J]. Journal of Midwifery and Women's Health, 2013, 58(1): 69-75.
[3] NCT 04441879. 2020. bBeAMom: Effectiveness of a Cognitive-Behavioral Blended Intervention for Postpartum Depression (bBeAMom). https://clinicaltrials.gov/study/NCT04441879
[4] NCT 05400161. 2022. Social Media-Based Parenting Program for Women With Postpartum Depressive Symptoms: Impact on Child Development. https://clinicaltrials.gov/study/NCT05400161

Reason 5 for exclusion: Conference abstracts(n = 4)

[1] MILGROM J, DANAHER B, SCHEMBRI C, et al. Web-based cognitive behavioural therapy for postnatal depression [J]. Archives of Women's Mental Health, 2013, 16(S48.
[2] O'MAHEN H, WILKINSON E, WOODFORD J, et al. The netmums project: A randomized controlled trial of online behavioural activation for postnatal depression [J]. Archives of Women's Mental Health, 2013, 16(S64.
[3] MILGROM J, DANAHER B, SEELEY J, et al. Mummoodbooster-an interactive internet treatment for postnatal depression [J]. Archives of Women's Mental Health, 2015, 18(2): 334-5.
[4] MILGROM J, DANAHER B G, GEMMILL A W, et al. Internet cognitive behavioural therapy for women with postnatal depression: A randomized controlled trial of MumMoodBooster [J]. Archives of Women's Mental Health, 2020, 23(2): 293-4.

Reason 6 for exclusion: Clinical registration trials with no available results(n = 5)

[1] GRIFFITHS K, CHRISTENSEN H, ELLWOOD D. Online cognitive behaviour therapy (MoodGYM) for the prevention of postnatal depression in at-risk mothers: a randomised controlled trial [J]. Australian new zealand clinical trials registry [wwwanzctrorgau], 2009
[2] NCT 05044455. 2021. Online Peer-Delivered Group CBT for PPD. https://clinicaltrials.gov/study/NCT05044455
[3] NCT 05269732. 2022. CBT for Postpartum Depression and Infant Emotion Regulation. https://clinicaltrials.gov/study/NCT05269732
[4] NCT 04045132. 2019. Social Media-Based Parenting Program for Women With Postpartum Depressive Symptoms. https://clinicaltrials.gov/study/NCT04045132
[5] NCT 05643898. 2022. "Mamá, te Entiendo" App-based Intervention for Reducing Depressive Symptoms in Postpartum Women. https://clinicaltrials.gov/study/NCT05643898

Reason 7 for exclusion: Ineligible outcome(n = 3)

[1] O'MAHEN H A, WILKINSON E, BAGNALL K, et al. Shape of change in internet based behavioral activation treatment for depression [J]. Behav Res Ther, 2017, 95(107-16.
[2] CHAUDHRY I, CHAUDHRY N, KHALID T, et al. Development and assessment of smartphone based intervention (TechMotherCare) for maternal depression in Pakistan [J]. Journal of Psychosomatic Research, 2020, 133(
[3] SEO J M, KIM S-J, NA H, et al. The Development of the Postpartum Depression Self-Management Mobile Application "Happy Mother" [J]. CIN: Computers, Informatics, Nursing, 2021, 39(8): 439-49.

Reason 8 for exclusion: Duplicates(n = 3)

[1] NGAI F W. Telephone-based cognitive-behavioral therapy on postnatal depression and quality of life [J]. BJOG: An International Journal of Obstetrics and Gynaecology, 2018, 125(18.
[2] FONSECA A, MONTEIRO F, ALVES S, et al. Be a Mom, a Web-Based Intervention to Prevent Postpartum Depression: The Enhancement of Self-Regulatory Skills and Its Association With Postpartum Depressive Symptoms [J]. Front Psychol, 2019, 10(265.
[3] BAGNALL K M. Long-term follow-up of NetmumsHWD: A feasibility randomised controlled trial of telephone supported online behavioural activation for postnatal depression at 16 months post-randomisation [D]; University of Exeter (United Kingdom), 2014.

Supplementary File S2 The titles of the included studies (N=18)

[1] MILGROM J, DANAHER B G, SEELEY J R, et al. Internet and Face-to-face Cognitive Behavioral Therapy for Postnatal Depression Compared With Treatment as Usual: Randomized Controlled Trial of MumMoodBooster[J/OL]. Journal of Medical Internet Research, 2021, 23(12): e17185. DOI:10.2196/17185.

[2] SEO J M, KIM S J, NA H, et al. Effectiveness of a Mobile Application for Postpartum Depression Self-Management: Evidence from a Randomised Controlled Trial in South Korea[J/OL]. Healthcare, 2022, 10(11): 2185. DOI:10.3390/healthcare10112185.

[3] BABIY Z, LAYTON H, SAVOY C D, et al. One-Day Peer-Delivered Cognitive Behavioral Therapy-Based Workshops for Postpartum Depression: A Randomized Controlled Trial[J/OL]. Psychotherapy and Psychosomatics, 2024, 93(2): 129-140. DOI:10.1159/000536040.

[4] CARONA C, PEREIRA M, ARAÚJO-PEDROSA A, et al. The Efficacy of Be a Mom, a Web-Based Intervention to Prevent Postpartum Depression: Examining Mechanisms of Change in a Randomized Controlled Trial[J/OL]. JMIR Mental Health, 2023, 10: e39253. DOI:10.2196/39253.

[5] CARONA C, PEREIRA M, ARAÚJO-PEDROSA A, et al. For Whom and for How Long Does the “Be a Mom” Intervention Work? A Secondary Analysis of Data From a Randomized Controlled Trial Exploring the Mid-Term Efficacy and Moderators of Treatment Response[J/OL]. Behavior Therapy, 2023: S0005789423001260. DOI:10.1016/j.beth.2023.11.001.

[6] FONSECA A, ALVES S, MONTEIRO F, et al. Be a Mom, a Web-Based Intervention to Prevent Postpartum Depression: Results From a Pilot Randomized Controlled Trial[J/OL]. Behavior Therapy, 2020, 51(4): 616-633. DOI:10.1016/j.beth.2019.09.007.

[7] JANNATI N, MAZHARI S, AHMADIAN L, et al. Effectiveness of an app-based cognitive behavioral therapy program for postpartum depression in primary care: A randomized controlled trial[J/OL]. International Journal of Medical Informatics, 2020, 141: 104145. DOI:10.1016/j.ijmedinf.2020.104145.

[8] MERZA D, AMANI B, SAVOY C, et al. Online peer-delivered group cognitive-behavioral therapy for postpartum depression: A randomized controlled trial[J/OL]. Acta Psychiatrica Scandinavica, 2023: acps.13611. DOI:10.1111/acps.13611.

[9] SHARIATPANAHI G, EFFATPANAH M, MOIENAFSHAR A, et al. Comparing the Effectiveness of Internet-Based Cognitive Behavioral Therapy and Drug Therapy for Treating Postpartum Depression and Children Weight Gain: A Randomized Clinical Trial[J/OL]. International Journal of High Risk Behaviors and Addiction, 2023, 11(4)[2024-04-23]. https://brieflands.com/articles/ijhrba-117830.html. DOI:10.5812/ijhrba-117830.

[10] VAN LIESHOUT R J, LAYTON H, SAVOY C D, et al. Effect of Online 1-Day Cognitive Behavioral Therapy–Based Workshops Plus Usual Care vs Usual Care Alone for Postpartum Depression: A Randomized Clinical Trial[J/OL]. JAMA Psychiatry, 2021, 78(11): 1200. DOI:10.1001/jamapsychiatry.2021.2488.

[11] HUH K, LAYTON H, SAVOY C D, et al. Online Public Health Nurse–Delivered Group Cognitive Behavioral Therapy for Postpartum Depression: A Randomized Controlled Trial During the COVID-19 Pandemic[J/OL]. The Journal of Clinical Psychiatry, 2023, 84(5)[2024-04-23]. https://www.psychiatrist.com/jcp/online-nurse-delivered-group-cbt-postpartum-depression. DOI:10.4088/JCP.22m14726.

[12] LOUGHNAN S A, BUTLER C, SIE A A, et al. A randomised controlled trial of ‘MUMentum postnatal’: Internet-delivered cognitive behavioural therapy for anxiety and depression in postpartum women[J/OL]. Behaviour Research and Therapy, 2019, 116: 94-103. DOI:10.1016/j.brat.2019.03.001.

[13] MILGROM J, DANAHER B G, GEMMILL A W, et al. Internet Cognitive Behavioral Therapy for Women With Postnatal Depression: A Randomized Controlled Trial of MumMoodBooster[J/OL]. Journal of Medical Internet Research, 2016, 18(3): e54. DOI:10.2196/jmir.4993.

[14] *NGAI F W, WONG P W C, LEUNG K Y, et al. The Effect of Telephone-Based Cognitive-Behavioral Therapy on Postnatal Depression: A Randomized Controlled Trial[J/OL]. Psychotherapy and Psychosomatics, 2015, 84(5): 294-303. DOI:10.1159/000430449.

[15] O’MAHEN H A, WOODFORD J, MCGINLEY J, et al. Internet-based behavioral activation—Treatment for postnatal depression (Netmums): A randomized controlled trial[J/OL]. Journal of Affective Disorders, 2013, 150(3): 814-822. DOI:10.1016/j.jad.2013.03.005.

[16] O'MAHEN H A, RICHARDS D A, WOODFORD J, et al. Netmums: a phase II randomized controlled trial of a guided Internet behavioural activation treatment for postpartum depression[J/OL]. Psychological Medicine, 2014, 44(8): 1675-1689. DOI:10.1017/S0033291713002092.

[17] PUGH N E, HADJISTAVROPOULOS H D, DIRKSE D. A Randomised Controlled Trial of Therapist-Assisted, Internet-Delivered Cognitive Behavior Therapy for Women with Maternal Depression[J/OL]. PLOS ONE, 2016, 11(3): e0149186. DOI:10.1371/journal.pone.0149186.

[18] *WOZNEY L, OLTHUIS J, LINGLEY-POTTIE P, et al. Strongest FamiliesTM Managing Our Mood (MOM): a randomized controlled trial of a distance intervention for women with postpartum depression[J/OL]. Archives of Women's Mental Health, 2017, 20(4): 525-537. DOI:10.1007/s00737-017-0732-y.

denotes included in systematic review only

Supplementary File S3 The complete process of contacting the original authors for clarification or data provision

List of references for contacting the original authors:

(Emails were sent to the corresponding author on May,13 2024 and May,20 2024, respectively)

[1]NGAI F W, WONG P W C, LEUNG K Y, et al. The Effect of Telephone-Based Cognitive-Behavioral Therapy on Postnatal Depression: A Randomized Controlled Trial[J/OL]. Psychotherapy and Psychosomatics, 2015, 84(5): 294-303. DOI:10.1159/000430449.

[2]WOZNEY L, OLTHUIS J, LINGLEY-POTTIE P, et al. Strongest FamiliesTM Managing Our Mood (MOM): a randomized controlled trial of a distance intervention for women with postpartum depression[J/OL]. Archives of Women's Mental Health, 2017, 20(4): 525-537. DOI:10.1007/s00737-017-0732-y.
